# SpeechDETECT: an explainable automated speech processing pipeline for early detection of neurological and health changes

**DOI:** 10.1007/s13755-026-00468-5

**Published:** 2026-07-24

**Authors:** Maryam Zolnoori, Elyas Esmaeili, Mehdi Naserian, Ali Zolnour, Sina Rashidi, Tahoura Morovati, Hossein Azadmaleki, Zhihong Zhang, James M. Noble, Margaret V. McDonald

**Affiliations:** 1 Columbia University Irving Medical Center, New York, NY 10027 USA; 2 School of Nursing, Columbia University, New York, NY 10027 USA; 3 Data Science Institute, Columbia University, New York, NY 10027 USA; 4 Department of Neurology, Taub Institute for Research on Alzheimer’s Disease and the Aging Brain, GH Sergievsky Center, Columbia University, New York, NY 10032 USA; 5 Center for Home Care Policy & Research, VNS Health, New York, NY 10017 USA; 6 Columbia University Irving Medical Center, 560 W 168 St, New York, NY 10032 USA; 7 Independent Researcher, New York, USA

## Abstract

**Background:**

Early detection of cognitive impairment remains a critical public health challenge. While biomarkers such as neuroimaging and cerebrospinal fluid analyses offer high sensitivity, their limited accessibility hampers widespread screening, especially in underserved settings. Speech-based markers have emerged as promising, noninvasive indicators of cognitive decline.

**Objective:**

To develop and validate SpeechDETECT, an end-to-end speech-processing pipeline that captures fine-grained acoustic and temporal markers of cognitive impairment and provides interpretable outputs suitable for large-scale screening.

**Methods:**

SpeechDETECT comprises six modules: (1) noise reduction / amplitude normalization; (2) an eight-domain voice-analysis framework (e.g., frequency parameters, speech fluency); (3) 50 ms segment-level feature extraction; (4) feature visualization; (5) dimensionality reduction / selection (Joint Mutual Information Maximization, LassoNet, PCA); and (6) classifier training with SHapley Additive exPlanations (SHAP). Performance was benchmarked against six acoustic toolkits (e.g., GeMAPS) on two English datasets: the DementiaBank Pitt corpus (train = 166, test = 71) with single cookie-theft picture description task and NIA PREPARE Phase 2 corpus (train = 1 064, test = 267) with multiple speech tasks.

**Results:**

A Multi-Layer Perceptron trained on PCA-derived SpeechDETECT features achieved an F1-score = 0.81% and AUC-ROC = 0.80 on the Pitt test set, outperforming the best competing toolkit (AUC = 0.76). On the PREPARE test set—comprising ≤ 30 s recordings from four speech tasks—the same model attained F1 ≈ 0.67% and AUC-ROC = 0.70**,** demonstrating good generalizability. Cumulative-gains analysis showed that screening the top 40% of ranked participants captured ~ 70% of cognitively-impaired (CI) cases in Pitt and ~ 63% in PREPARE. SHAP revealed speech-fluency metrics (hesitation rate, pause ratio) and high-frequency formant dynamics as the most discriminative features.

**Conclusion:**

SpeechDETECT delivers accurate (AUC up to 0.80) and interpretable detection of early cognitive impairment across both structured and multi-task speech settings. Its fully automated, domain-informed approach enables scalable, speech-based screening and provides a foundation for multimodal systems that combine acoustic markers with clinical or biomarker data to further improve diagnostic precision*.* The SpeechDETECT toolkit is openly available on GitHub at https://github.com/SpeechCARE/SpeechDETECT-Toolkit for researchers and clinicians. A demo tutorial video showing pipeline usage is available at https://github.com/SpeechCARE/SpeechDETECT-Toolkit/blob/main/SpeechDETECT.mp4.

## Introduction

Alzheimer’s disease and related dementias (ADRD) are rising public health concerns. One in five older adults is at significant risk for or already has cognitive impairment [1–4]. Despite nationwide efforts and the availability of sensitive biomarkers (e.g., MRI and CSF), more than 50% of patients with early cognitive changes remain undiagnosed and untreated.[5–7] This is mainly due to patients’ inability to recognize or distinguish early symptoms from normal cognitive aging [8], clinicians’ time constraints for evaluation, [9, 10] and limited access to specialists and diagnostic tools, particularly among minoritized groups [11–14]. These barriers delay diagnosis, negatively affect daily functioning and quality of life, and complicate healthy aging [7, 15], with costs projected to exceed $1 trillion by 2050. In the era of increasing biomarker diagnostics and disease-modifying therapies for Alzheimer’s disease, timely clinical identification is critical.

**Fig. 1:**
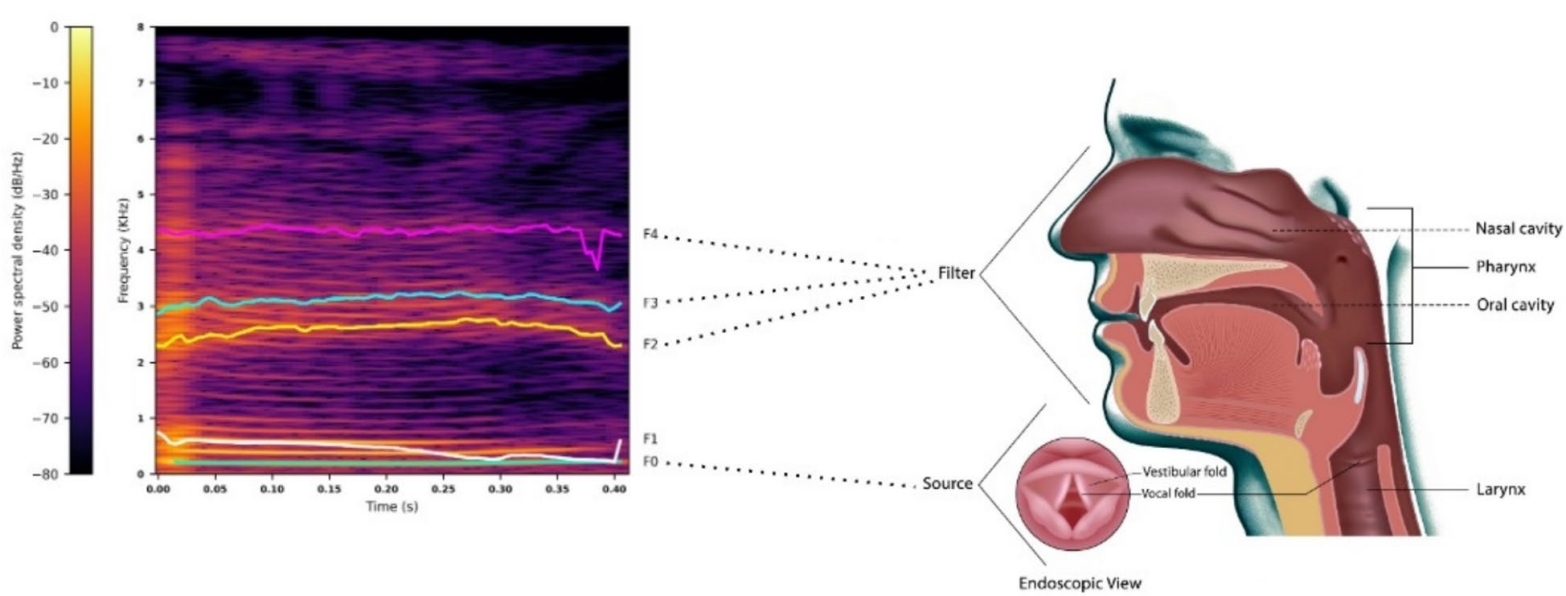
Speech production in human applies the source-filter framework, where the ‘source’ in the larynx creates vocalizations. This is achieved as air exhaled from the lungs vibrates the vocal folds, setting the fundamental frequency of the sound (F0). The sound waves then pass through the vocal tract, including the pharynx, oral and nasal cavities, where they are filtered—enhancing some frequencies and attenuating others to produce resonant frequencies known as formants (F1, F2, F3, F4). This filtering varies with the vocal tract’s characteristics, like cavity shape and size, and tongue position. These frequency parameters are prominently displayed in the spectrogram profile of the voice, illustrating how they change over time and providing detailed view of an individual’s speech dynamics. This entire process is demonstrated in this figure, corresponding to the pronunciation of the vowel /ɑ/

Emerging studies show that speech impairment is one of the earliest signs of cognitive decline, making spoken language a biomarker for multiple dimensions of cognitive abilities, including executive functioning, semantic memory, and language [16–20]. Speech production in humans follows the source-filter framework [21] (Fig. 1). The source, located in the larynx, generates sound through vocal fold oscillations as air is exhaled from the lungs, determining the fundamental frequency (F0) [22]. The sound then travels through the vocal tract, including the pharynx, oral, and nasal cavities, where it is filtered, enhancing or reducing frequencies to produce Formant Frequencies [23] (F1, F2, F3). This filtering varies with the vocal tract’s characteristics, such as shape, size, and tongue position. These frequency parameters are prominently displayed in the spectrogram profile of the voice—a visual representation illustrating variations in voice frequency and intensity over time (Fig. 1)— which provides a detailed view of an individual’s speech dynamics. In individuals with cognitive impairments, control over vital speech organs, particularly the vocal folds and tongue, is compromised [24] This can be documented by measuring acoustic characteristics, such as the point, mode, and tension of the vocal tract, reflecting the patient’s ability to control vocal organs.

The speech processing pipelines built on the acoustic component of speech for detecting cognitive impairment typically employ two approaches: (1) hand-crafted acoustic features, analyzing sets of acoustic and linguistic features such as Formant Frequencies and Pause Rate, often using established acoustic repository sets like GeMAPS [25] tailored for emotion recognition; (2) Deep learning architectures like YAMNet [26] and VGGish [27], which autonomously learn representations from voice spectrograms (Fig. 1). While these approaches capture acoustic cues linked to cognitive impairment, their performance —accuracy ranging from 54 to 77.7%–is suboptimal and offers limited explainability into how speech production mechanisms are affected by cognitive impairment.

To address these limitations, this study developed "SpeechDETECT," an integrated and explainable acoustic pipeline designed to detect speech cues indicative of cognitive impairment. Unlike existing methods that focus on a narrow subset of acoustic-linguistic features or rely on black-box deep learning alone, SpeechDETECT integrates domain-informed parameters spanning multiple dimensions of vocal production, thereby enhancing both interpretability and sensitivity to subtle cognitive changes. SpeechDETECT evaluates vocal traits across eight domains—including Frequency Parameters, Cepstral Coefficients and Spectral Features, Voice Quality, Loudness and Intensity, and Speech Signal Complexity—and temporal aspects, including Speech Fluency, Rhythmic Structure, and Speech Production Dynamics.

We assessed SpeechDETECT performance using two complementary English-language datasets: the DementiaBank Pitt Corpus (Fig. 8), consisting of structured picture-description speech, and the Pioneering Research for Early Prediction of Alzheimer’s and Related Dementias (PREPARE) Phase 2 Challenge dataset, which includes diverse short speech tasks such as Cookie-Theft, story recall, semantic fluency, and Alexa-based dialogue. By applying machine learning classifiers, SpeechDETECT effectively captured speech patterns indicative of early cognitive decline across both datasets. Compared to widely used acoustic feature toolkits such as GeMAPS [25] (Geneva Minimalistic Acoustic Parameter Set) and ComParE [28, 29] (Computational Paralinguistics Challenge, 2013/2016), SpeechDETECT provides a validated, domain-informed framework for analyzing speech-related markers into speech-related cognitive markers, supporting improved early detection of cognitive impairment.

The remainder of this paper is organized as follows: Sect. "Related work" reviews related work on acoustic features and deep learning approaches for speech-based detection of cognitive impairment. Sect. "Methodology" details the SpeechDETECT pipeline, including dataset characteristics, feature extraction, and machine learning models. Sect. "Results" presents the experimental results and SHAP-based explainability findings. Sect. "Discussion" discusses the implications, limitations, and future directions. Finally, Sect. "Conclusion" concludes the study.

## Related work

Early work on voice-based detection of cognitive impairment largely relied on hand crafted acoustic feature sets. These sets, such as prosodic, spectral, and voice quality parameters, were originally developed for tasks like affect recognition or speaker characterization but were later adapted for dementia screening [30–32]. More recent studies shifted toward neural architectures and embedding models trained directly on speech recordings, including convolutional and recurrent networks as well as pre-trained acoustic models. These models can capture subtle linguistic cues expressed through vocal patterns, but they typically require large datasets for fine-tuning to achieve robust performance [31, 33, 34]. This remains a major challenge in cognitive impairment research, as dataset growth is hindered by privacy regulations, restrictions on data sharing, and clinician reluctance to collect audio [35].

**Fig. 2:**
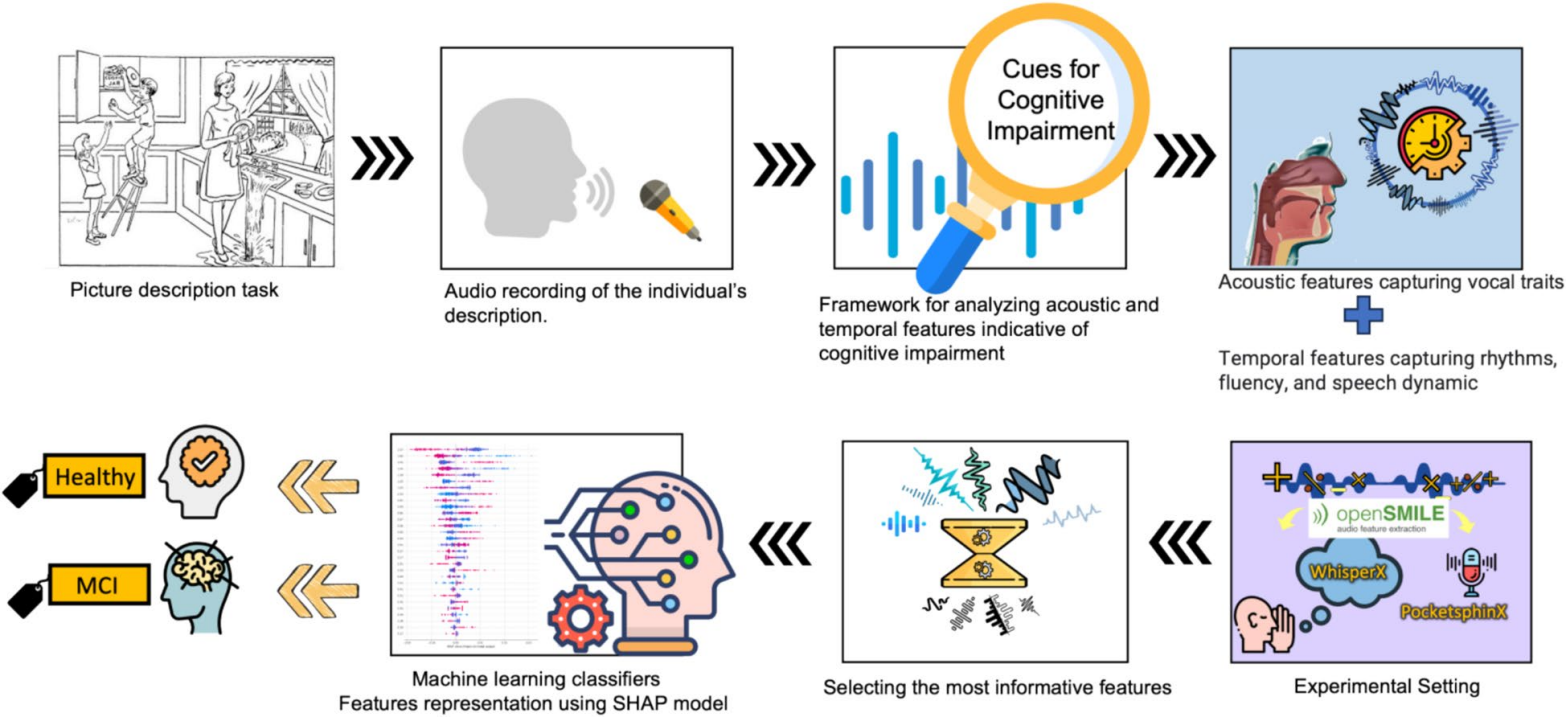
an overview of the SpeechDETECT pipeline

Given these constraints, most studies have relied on the DementiaBank Pitt corpus (see “Datasets” section), which provides standardized audio recordings of participants describing the Cookie-Theft picture (Fig. 2). Each participant underwent thorough neurological and neuropsychological assessments, with diagnoses confirmed by cognitive impairment experts (Appendix B details inclusion and exclusion criteria). In contrast, the NIA PREPARE Challenge dataset, released in late 2024, is newly available and, to date, has no published studies. Therefore, the literature review below focuses on the benchmark DementiaBank Pitt corpus**,** specifically highlighting studies that analyzed the acoustic component of voice to provide a baseline for evaluating SpeechDETECT**.**

Most prior studies [30–32, 36] using the DementiaBank Pitt corpus applied hand-crafted acoustic features extracted from toolkits such as AVEC (Audio/Visual Emotion Challenge, 2013), ComParE [28, 29] (Computational Paralinguistics Challenge, 2013/2016), GeMAPS/eGeMAPS [25] (Geneva Minimalistic Acoustic Parameter Set), IS10-Paraling [37] (INTERSPEECH 2010 Paralinguistic Challenge), Emo-Base Large, and COVAREP [38] (COoperative Voice Analysis REpository for Pitch). While these toolkits capture vocal attributes such as frequency, spectral features, voice quality, and intensity, they were originally developed for tasks like speaker trait recognition, affective computing, or biological sound classification. Table 1 summarizes these studies; for example, one study [36] reported the highest F1-score (0.7711) using logistic regression trained on a combination of eGeMAPS, ComParE-2016, and IS10-Paraling features.

**Table 1:** Summary of studies analyzed acoustic component of voice on the publicly available Benchmark dataset. Studies are grouped by methodological type ("Hand-crafted features", "Hand-crafted features + Deep learning models") and then ordered within each category by publication year

Study	Year	Dataset	Methodology/ Models	Key Findings	Shortcomings	Performance
Hand-crafted features						
Martinc et al. [32]	2020	ADReSSo 2020 challenge	eGeMAPS plus temporal and spectral acoustic features; acoustic subset of a multimodal grid-search framework	Acoustic configuration achieved 0.57 accuracy	Limited acoustic feature scope; eGeMAPS was designed for affective analysis rather than dementia	Accuracy 0.57 F1 not reported
Shah et al. [30]	2021	DementiaBank database	AVEC-2013, ComParE-2013, and emo_large with MLP, AdaBoost, and Decision Tree models; majority-vote ensemble	Best acoustic ensemble achieved Accuracy 0.65 and F1 0.73	High feature overlap and redundancy; feature sets were not optimized for dementia-specific speech	Accuracy 0.65 F1 0.73
Chen et al. [36]	2021	ADReSSo 2021 challenge (speech only)	GeMAPS, eGeMAPS, ComParE-2016, IS10-Paraling, and MFCCs with logistic regression on combined features	Combined handcrafted features produced Accuracy 0.77 and F1 0.78	High dimensionality and redundancy; toolkits were not designed for cognitive-impairment detection	Accuracy 0.77 F1 0.78
Choi et al. [31]	2024	Cross-linguistic picture-description corpora (American English, Korean, Mandarin Chinese)	eGeMAPSv2 and ComParE on segmented and non-segmented audio; eight ML/DL classifiers	MLP on non-segmented ComParE features achieved the best performance (Accuracy 0.84; AUC 0.80; F1 0.74 in manuscript summary)	Predominantly English data within a multilingual setting; acoustic sets still adapted from non-dementia tasks	Accuracy 0.84 F1 0.74
Niemelä et al. [49]	2024	ADReSSo 2020 challenge	openSMILE/eGeMAPS features from voiced segments; bag-of-acoustic-words representation; Wilcoxon feature selection; χ^2^-kernel SVM	Consistent 0.75 accuracy with 25 selected acoustic features on external test and LOSO evaluation	Limited acoustic diversity; histogram quantization may lose temporal detail; features remain non-dementia-specific	Accuracy 0.75 Approx. F1 0.72
Hand-crafted features + deep learning models						
Pappagari et al. [50]	2020	ADReSSo 2020 challenge	MFCC and pause-related features plus x-vector embeddings; PLDA classifier	Achieved Accuracy 0.66 and F1 0.69	x-vectors were trained for speaker recognition rather than pathology, limiting transferability and interpretability	Accuracy 0.66 F1 0.69
Koo et al. [51]	2020	ADReSSo 2020 challenge	ComParE and eGeMAPS feature sets plus VGGish embeddings; CNN-BiLSTM models	VGGish configuration performed best (Accuracy 0.72; F1 0.69)	General-audio pretraining and a complex architecture increase overfitting risk on small dementia datasets	Accuracy 0.72 F1 0.69
Pompili et al.[45]	2020	ADReSSo 2020 challenge	x-vector and i-vector embeddings with SVM	Achieved Accuracy 0.54 and F1 0.54	Embeddings emphasize speaker identity more than pathology and provide limited interpretability	Accuracy 0.54 F1 0.54
Rohanian et al.[52]	2021	ADReSSo 2021 challenge	79 COVAREP acoustic features with LSTM-based modeling within a multimodal framework; acoustic result reported separately	Acoustic configuration achieved 0.64 accuracy	Handcrafted feature dependence persists, and LSTM models are prone to overfitting with limited clinical speech data	Accuracy 0.64 F1 not reported
Syed et al.[53]	2020	ADReSSo 2020 challenge	IS10-Paraling, ComParE-2016, COVAREP, and VGGish embeddings with SVM	Best acoustic model achieved 0.64 accuracy	Generic paralinguistic and general-audio features may miss dementia-specific cues	Accuracy 0.64 F1 not reported
Chlasta et al.[54]	2021	ADReSSo 2020 challenge	VGGish embeddings with DemCNN / conventional classifiers	Achieved Accuracy 0.62 and F1 0.62	VGGish pretraining targets audio-event detection, not cognitive-impairment speech	Accuracy 0.62 F1 0.62
Gauder et al.[33]	2021	ADReSSo 2021 challenge	eGeMAPS plus TRILL, Allosaurus, and wav2vec 2.0 embeddings; neural model over short speech segments	wav2vec 2.0 embeddings achieved the highest reported test accuracy (0.79)	High computational demand and clinically opaque learned representations	Accuracy 0.79 F1 not reported
Zhu et al. [55]	2021	ADReSSo 2020 challenge	Transfer learning with MobileNet, YAMNet, and SpeechBERT audio models	YAMNet was best among audio models (Accuracy 0.66; F1 0.68)	Pretrained models were built for general audio/language tasks and remain weakly interpretable	Accuracy 0.66 F1 0.68
Bertini et al.[56]	2022	English spontaneous-speech dementia corpus	auDeep unsupervised spectrogram embeddings plus MLP classification	Achieved Accuracy 0.73 and F1 0.62	Unsupervised embeddings were not tailored to dementia-related cues; interpretability remains limited	Accuracy 0.73 F1 0.62
Ilias et al.[57]	2023	ADReSSo 2021 challenge	Vision Transformer on MFCC and log-Mel spectrogram inputs	Best result on log-Mel input (Accuracy 0.65; F1 0.69)	ViT was adapted from image modeling and may underrepresent temporal speech structure	Accuracy 0.65 F1 0.69
Priyadarshinee et al. [34]	2023	English AD speech benchmark	eGeMAPS, Emobase, Emobase-Large, VGGish, and OpenL3 with BiLSTM	VGGish + BiLSTM achieved the best reported accuracy (0.78)	Generic audio features still dominate and disease-specific interpretability remains limited	Accuracy 0.78 F1 not reported
Kim et al. [58]	2024	Korean and English 30-s AD speech datasets	Handcrafted pitch/jitter/shimmer/spectral features with ML models; deep models including x-vector on raw waveforms and spectrograms	Deep model (x-vector) outperformed traditional acoustic models on English data (Accuracy 0.78; F1 0.79)	Handcrafted feature set was narrow, and x-vectors remain speaker-oriented and less interpretable	Accuracy 0.78 F1 0.79

Other studies employed deep learning models based on Convolutional Neural Networks (CNNs) and Long Short-Term Memory (LSTM) networks to analyze voice spectrograms. Models such as MobileNet [39], YAMNet, [26] VGGish [40], X-vector [41], and i-vector [42, 43] have been used to extract acoustic embeddings. However, many were trained for unrelated tasks—event classification (e.g., YouTube audio in YAMNet and VGGish) or speaker identification (e.g., VoxCeleb [44] for X-vector)—limiting their ability to detect subtle vocal cues of cognitive decline. For instance, one study [45] reported an F1-score of 0.54 using X-vector and i-vector embeddings.

Recent advances include transformer-based models such as Wav2vec 2.0 [46] and SpeechBERT [47], which leverage attention mechanisms [48] to capture long-range dependencies and generate context-aware speech embeddings. While these models offer improved representational power, their high computational demands and need for large-scale fine-tuning have limited practical use. Notably, one study [33] achieved the highest reported accuracy (0.78) on this dataset using Wav2vec 2.0 (see Table 1).

To address the limitations of prior methods, we developed SpeechDETECT, an integrated speech-analysis pipeline that analyzes speech across multiple acoustic and temporal domains (Components 2–1 through 2–8 in the Methodology section). Unlike conventional models designed for emotion or speaker recognition, SpeechDETECT is tailored to identify fine-grained vocal indicators of cognitive impairment, offering enhanced interpretability and sensitivity. On the DementiaBank dataset, SpeechDETECT outperformed prior approaches focused on the acoustic component of voice for cognitive impairment detection.

## Methodology

This section describes the design and implementation of SpeechDETECT, an end-to-end acoustic pipeline for detecting cognitive impairment from speech. The pipeline is grounded in established principles from speech science, acoustic signal processing, and statistical learning, which support the use of frequency structure, spectral shape, voice quality, and temporal organization as informative representations of speech production. Guided by this literature, SpeechDETECT transforms raw recordings into interpretable acoustic and temporal markers through a sequence of preprocessing, feature extraction, feature refinement, and supervised classification steps. After introducing the overall structure of the pipeline, we describe each component, followed by the datasets, baseline acoustic toolkits, and analytical procedures used for evaluation.

### Overview of the SpeechDETECT architecture

SpeechDETECT is an integrated and explainable acoustic analysis pipeline that converts raw speech recordings into structured, clinically interpretable representations for detecting cognitive impairment. The architecture is organized as a sequence of processing stages that progressively standardize the audio, derive multidomain acoustic and temporal measures, summarize these measures across speech segments, and refine them for classification. Its design is guided by evidence that cognitive decline can affect multiple dimensions of speech production, including vocal control, prosody, articulation, fluency, and temporal organization; accordingly, the pipeline captures features related to fundamental frequency, formant structure, spectral properties, voice quality, loudness, rhythmic timing, and speech production dynamics. Rather than treating speech as a single undifferentiated signal, SpeechDETECT preserves intermediate representations, such as spectrograms, temporal trajectories, and distributional summaries, that support qualitative inspection and systematic error analysis. This structure allows the pipeline to remain both analytically rigorous and physiologically interpretable while supporting downstream machine-learning models for classification.

Figure 2 provides a high-level overview of the SpeechDETECT workflow, which converts raw audio recordings into interpretable acoustic and temporal representations for classification. In Component 1, raw recordings are standardized through denoising, amplitude normalization, and waveform formatting to reduce recording-related variability. In Component 2, the cleaned signal is mapped to an eight-domain analytical framework that organizes acoustic information into physiologically and clinically meaningful categories. In Component 3, each recording is divided into short overlapping segments, and segment-level descriptors are computed and aggregated into subject-level acoustic and temporal features. In Component 4, these extracted features are visualized through spectrograms, trajectories, and distributional summaries to support qualitative inspection. In Component 5, the high-dimensional feature matrix is transformed through selection and dimensionality-reduction methods to remove redundancy and retain the most informative structure. In Component 6, the reduced feature representation is used to train and evaluate supervised machine-learning models, and SHAP is applied to quantify feature contributions to model predictions.

The following subsections provide detailed descriptions of each component of the SpeechDETECT pipeline and the datasets, baseline acoustic toolkits, and analytical procedures used in this study.

#### Component 1- audio preprocessing: noise reduction and normalization

The recorded speech contained stationary noise from the recording device and environmental sources, such as air conditioning or background hums. To address this, a low-pass filter (LPF) was applied using SciPy, a Python-based library, for noise reduction. Amplitude normalization was subsequently performed with NumPy, scaling the range to -1 to 1 to accommodate variations in recording setups, such as differences in speaker positions and microphone distances. The preprocessed audio was saved as 16-bit mono-channel.wav files, preserving the original sample rate of 44,100 Hz.

#### Component 2–voice analysis framework: characterizing vocal traits and temporal dynamics

We developed this framework by reviewing 44 studies retrieved from PubMed, Web of Science, and Scopus that explored spontaneous speech tasks—picture description, storytelling, and semi-structured dialogues—combined with machine learning techniques for early detection of cognitive impairment. From these studies, we synthesized key acoustic and temporal features linked to cognitive decline, ultimately defining eight primary domains: **frequency parameters**, **Cepstral Coefficients and Spectral Features, voice quality**, **loudness and intensity**, **speech signal complexity**, **rhythmic structure, speech fluency, and speech production dynamics.**

**Fig. 3:**
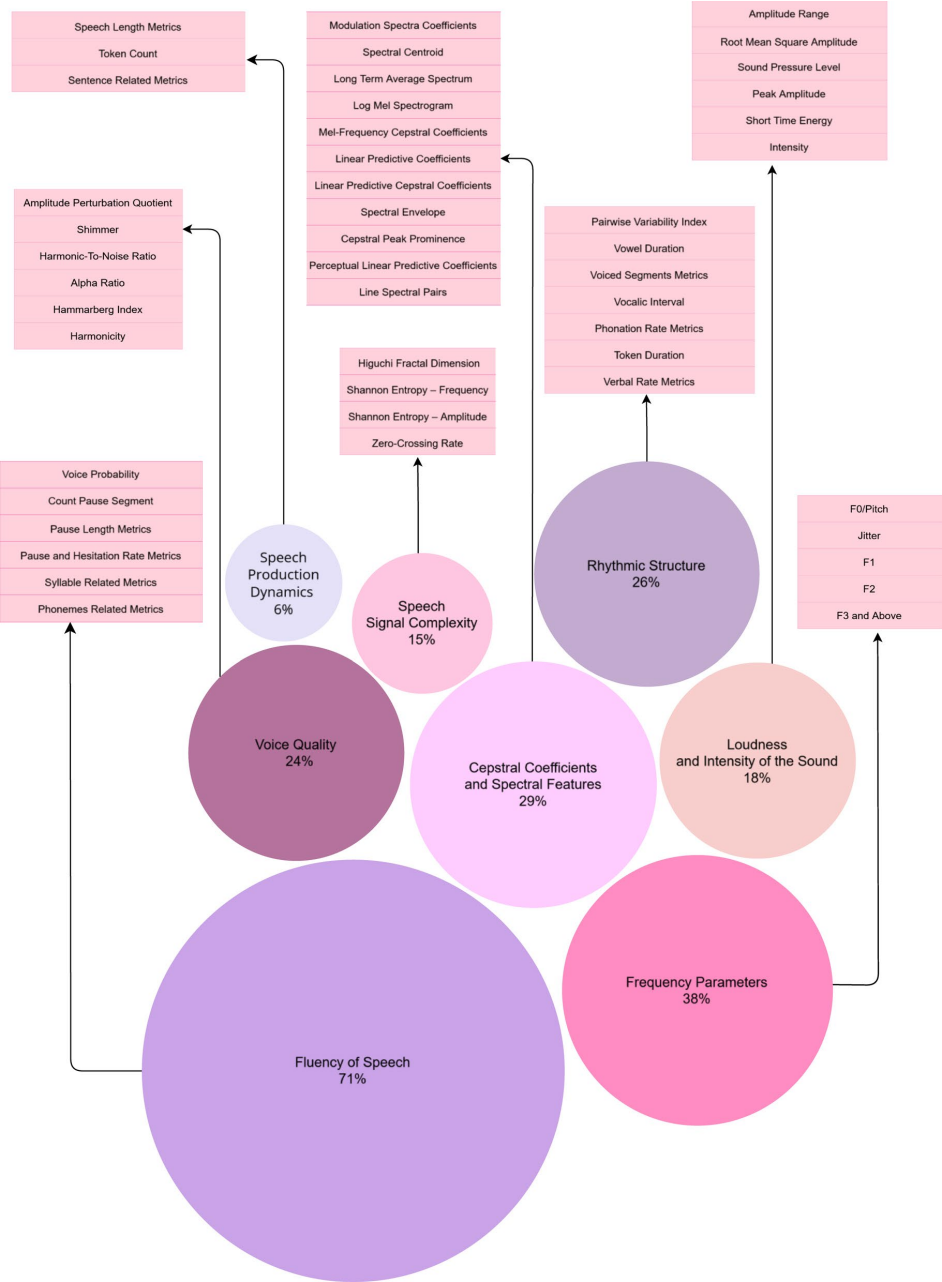
The eight acoustic domains and their corresponding features

Figure 3 illustrates these eight acoustic domains, arranged as circles of varying sizes to represent the frequency of their use across the reviewed literature. The percentages inside each circle indicate how many studies examined that domain, offering a quick visual comparison of prevalence. Each domain encompasses specific acoustic/temporal features that map to broader categories of speech production. Notably, speech fluency (the largest circle) appeared most often (71%, 25 studies reporting significance), whereas speech signal complexity (the smallest circle) was the least utilized (15%, 5 studies reporting significance). Components 2.1 through 2.8 provide a concise overview of representative features within each domain and detail how these features relate to cognitive impairment. For more in-depth explanations of the acoustic domains and their corresponding features, please refer to Table 7**.**

#### Component 2.1. frequency features

Frequency parameters, including fundamental frequency [22] (F0) and formant frequencies [23] (F1, F2, F3, F4), are key to conveying information on vocal fold vibrations and vocal tract configurations during speech. F0, the lowest frequency in the speech signal, determines perceived pitch, while formants reflect the acoustic properties of the vocal tract. In cognitive impairment, compromised motor planning and coordination increase F0 variability, leading to irregular pitch [59]. Cognitive decline also affects the precise control of speech organs, (e.g., tongue) required for phoneme articulation, altering formant frequencies [60]. High-frequency formants, particularly F3 (1500–2500 Hz), are most impacted, making vowel and syllable articulation more challenging. These disruptions reduce speech clarity and consistency, highlighting difficulties in the cognitive regulation of motor speech processes.

#### Component 2.2. cepstral coefficients and spectral features

Cepstral Coefficients and Spectral features describe the voice’s spectral shape and dynamics by analyzing the distribution of energy across frequency components over time [61] (refer to the spectrogram in Fig. 1). These features reveal how the voice is projected and perceived. Features such as the Spectral Centroid assesses the spectrum’s center of mass, indicating the average frequency where energy concentrates and perceived brightness [62]. Mel-frequency Cepstral Coefficients [61] (MFCCs) mimic human auditory processing by capturing spectral features on the mel scale. Additionally, the Long-Term Average Spectrum [63] (LTAS) provides insights into vocal stability by analyzing energy distribution across frequencies over time. In cognitive impairment, diminished motor control alters these features: the Spectral Centroid shifts to lower frequencies, MFCCs show reduced complexity, and LTAS reveals decreased variability, resulting in monotonous and slurred speech patterns [24].

#### Component 2.3. voice quality

Voice quality refers to the distinctive characteristics of a speaker’s voice that shape how speech is perceived [64]. Examples include the Noise-to-Harmonic Ratio [65] (NHR), which quantifies the proportion of noise (aperiodic) to harmonic (periodic) sound, reflecting perceived noisiness; Shimmer [66], which measures amplitude variation between consecutive vocal cycles, indicating voice stability; and the Acoustic Voice Quality Index [67] (AVQI), which combines time- and frequency-domain metrics to assess overall voice quality. In cognitive impairment, studies show that due to impaired coordination and motor control NHR increases, indicating more noise in the voice signal [68]. Shimmer values rise, suggesting greater amplitude variability, while higher AVQI scores reflect poorer voice quality and pronounced changes in time and frequency characteristics [69].

#### Component 2.4. loudness and intensity

Intensity [70] measures the energy of a sound wave, while loudness [71] is the subjective perception of sound pressure, both influenced by the structure of vocal organs and neural control of vocal folds. Effective modulation of intensity requires precise neural coordination. Examples of features for measuring intensity are Sound Pressure Level [72] (SPL), quantifying acoustic energy relative to the lowest hearing threshold (20 µPa ~ 0 dB); Root Mean Square (RMS) Amplitude, which calculates the square root of the average squared amplitude values to measure overall sound energy; and Short Time Energy [73], assessing the average power of a sound signal over short segments to capture dynamic intensity changes. In cognitive impairment, diminished control over intensity often results in monotonous, less articulate speech [24].

#### Component 2.5. voice signal complexity

Speech signal complexity analyzes variations in frequency and amplitude and their interactions over time. Features such as Multiscale Permutation Entropy [74] (MPE) and Shannon Entropy [75] measure the variability and unpredictability of speech signals, with lower values indicating reduced diversity and greater predictability. Similarly, Higuchi’s Fractal Dimension [76] (HFD) evaluates the irregularity of the speech signal. In cognitive impairment, speech complexity typically decreases due to impaired neural coordination. This decline affects the patient’s ability to modulate tone and maintain rhythm, resulting in more predictable and simplified speech patterns [76].

#### Component 2.6. rhythmic structure

Rhythm [77] refers to the systematic arrangement of phonemes—the smallest sound units that distinguish words (e.g., "p" and "b" in "pat" vs. "bat")—into syllables and words, characterized by timing, duration, and sequential patterns. Effective rhythm relies on precise phoneme production, where articulatory movements coordinate to produce clear, temporally structured speech. In cognitive impairment, deficits in phonetic-motor planning disrupt the articulation of phonemes, leading to inconsistencies in their duration, clarity, and timing [78]. These disruptions affect the temporal alignment of syllables and words, compromising rhythm and speech intelligibility. Quantitative measures of these changes include extended vowel durations [79] (indicating slower articulatory transitions), the Pairwise Variability Index [80] (capturing variability in syllable timing), and irregular voiced segment durations [81] (highlighting inconsistencies in phoneme production). Additionally, imprecise articulation of phonemes can result in blurred syllable boundaries and reduced distinctiveness in word formation [81]. As cognitive impairment progresses, rhythmic disruptions intensify, with speech becoming increasingly monotonous and characterized by tremors or unstable vocal patterns [78].

#### Component 2.7. speech fluency

Speech fluency [82] is characterized by the smooth, uninterrupted flow of speech. In patients with cognitive impairment, fluency deteriorates, evidenced by increased pauses and a higher frequency of pauses per spoken token, primarily due to difficulties in retrieving vocabulary and organizing thoughts [82]. This deterioration can be measured using silent pauses, filler pauses (e.g., non-lexical fillers like "um"), hesitation rate (frequency of filler pauses), within-word disfluency(e.g., silent pauses within words or prolonged phonemes), and voice breaks (disruptions in voice continuity) [83]. As cognitive impairment progresses, these disruptions become more pronounced, resulting in fragmented and less coherent speech [84].

#### Component 2.8. speech production dynamics

Speech production dynamics [85] reflect the overarching patterns of speech delivery, including the rate, length, and complexity of spoken output**.** This component evaluates how efficiently speech is structured and delivered, providing insights into an individual’s linguistic proficiency and communicative competence. Unlike rhythm and fluency, which address micro-level features like timing and pauses, production dynamics emphasize macro-level characteristics**,** such as utterance length, speech rate, and token density. In patients with cognitive impairment, deficits in working memory and executive function disrupt the ability to formulate complex thoughts and retrieve vocabulary, resulting in shorter, simpler utterances, slower speech, and increased reliance on fragmented phrases [86]. Examples of metrics for quantifying these disruptions include Relative Sentence Duration**,** Mean Sentence Length, and the Total Number of Tokens. As cognitive impairment progresses, these macro-level patterns deteriorate further, contributing to less efficient and increasingly fragmented communication [87].

#### Component 3- computing acoustic and temporal features

To compute acoustic features representing vocal traits—including Frequency Parameters, Cepstral Coefficients, Spectral Features, Voice Quality, Loudness, Intensity, and Speech Signal Complexity—we segmented each subject’s speech into 50-ms (ms) segments. This length captures subtle spectrogram fluctuations, enabling reliable assessment of vocal traits linked to cognitive decline. Shorter segments often miss critical features like vowel articulation or phoneme transitions, especially in older adults with slower, more variable vocal traits. We applied a 50% overlap between consecutive segments to preserve continuity, a standard practice in speech processing that minimizes loss of contextual data at segment boundaries.

To aggregate acoustic features across speech segments, we used a comprehensive set of statistical functions (e.g., mean, standard deviation, linear regression coefficients, quartiles) provided by the OpenSMILE library [88] —an open-source tool for signal processing and machine learning. Details of these functions are in Table 8**.**

To analyze temporal speech features—including fluency, rhythmic structure, and production dynamics—we employed a multi-step process combining WhisperX [89], Pocketsphinx [90], and the CMUDICT toolkit. WhisperX, an open-source ASR system based on OpenAI’s Whisper model, was used to generate word-level timestamps, offering high precision due to its encoder-decoder architecture and training on extensive labeled datasets. These word boundaries were passed to Pocketsphinx, a lightweight tool optimized for phoneme identification, to generate phoneme-level timestamps. The word and phoneme boundaries were subsequently integrated using the CMUDICT toolkit to achieve accurate syllable delineation. This workflow ensured efficient computation of key temporal features, including syllabic interval duration [24], token duration, vowel duration, and speech rate (**see details in **Table 7).

The outcome of this component is a detailed list of acoustic and temporal features used for training machine learning algorithms.

#### Component 4 –visualization of acoustic and temporal features

Following feature extraction, SpeechDETECT provides a visualization capability to display specific vocal characteristics within a selected utterance or speech segment, enabling direct comparisons between CI and CN speakers. For illustrative purposes, only a subset of features and a short-spoken sentence are showcased in the Results section.

#### Component 5- feature selection

To avoid overfitting when constructing machine learning (ML) classifiers with all acoustic features, we employed feature selection techniques. We evaluated four distinct methods: JMIM [91] (Joint Mutual Information Maximization), which selects features maximizing mutual dependence with the outcome while minimizing redundancy; LASSO (Least Absolute Shrinkage and Selection Operator), which uses an L1 penalty to shrink irrelevant feature coefficients to zero; LassoNet [92], an extension of LASSO [93] that integrates a multilayer perceptron (MLP) to capture nonlinear relationships and handle data sparsity; and Principal Component Analysis [94] (PCA), which reduces dimensionality by transforming data into orthogonal components while preserving variability. Details of these methods are provided in Appendix E.

#### Component 6- building machine learning (ML) classifiers

##### ML classifiers

We evaluated the performance of various ML classifiers, including traditional models and a Multi-Layer Perceptron [95] (MLP). Traditional classifiers included Support Vector Machine [96] and ensemble tree-based methods (Random Forest [97], XGBoost, AdaBoost [98], and Extra Trees [99]), widely used in health studies for their effectiveness with small samples and high-dimensional feature sets. Advanced deep learning models, such as Bi-LSTM [100] and CNN, [101] were excluded due to the study’s small sample size and the risk of overfitting associated with their complex architectures. Hyperparameter tuning for each model (Table 9) was conducted using 5-fold cross-validation, with the F1 score as the optimization metric.

##### Evaluation metrics

To evaluate pipeline performance on the test dataset, we used standard metrics, including AUC-ROC, precision-recall curve, cumulative gain curve, sensitivity, positive predictive value (PPV), and F1-score (the harmonic mean of precision and recall) across all classification thresholds. AUC-ROC, which represents the tradeoff between True Positive Rate (TPR or sensitivity) and False Positive Rate (FPR or 1-specificity), is widely regarded as a benchmark metric. Its robustness to class imbalance and consistent evaluation of binary classification models makes it a reliable measure of overall model performance.

### Datasets

In this study we use two datasets: DementiaBank‑Pitt Corpus (Table 2) and PREPARE Phase 2 (Table 3) to evaluate SpeechDETECT for early cognitive‑decline detection. To our knowledge, no prior work has applied a structured speech-analysis pipeline to the PREPARE Phase 2 corpus. Baseline models and prior benchmark results on these datasets are summarized in Sect. "Related work" (Related Work) and Table 1; here we describe the datasets used to evaluate SpeechDETECT pipeline.

**Table 2:** Participants characteristics of pitt corpus dataset

Development (Training) Dataset											
Attributes	Cognitively impaired participants (CI)					Cognitively normal participants (CN)					
	N = 87 (F/M = 58/29)					N = 79 (F/M = 52/27)					
	Mean ± Std	Range	Quartile 25%	Quartile 50%	Quartile 75%	Mean ± Std	Range	Quartile 25%	Quartile 50%	Quartile 75%	P-Value
Age	69.72 ± 6.8	53 – 80	65	70	75	66.04 ± 6.25	54–80	62	66	71	0.00
MMSE score	17.44 ± 5.33	3 – 28	14	18	20	28.99 ± 1.15	26–30	28	29	30	0.00
Audio length	87.61 ± 46.58	35.26 – 268.49	56.42	76.35	100.17	68.76 ± 25.57	22.79–168.61	52.01	67.77	78.38	0.01
Speech rate	78.80 ± 31.92	18.0 – 166.31	57.5	78.02	98.06	105.08 ± 29.34	46.19–175.24	87.80	100.07	123.66	0.00
Pause ratio	0.57 ± 0.2	0.10 – 0.96	0.42	0.55	0.76	0.42 ± 0.18	0.18–0.95	0.28	0.40	0.53	0.00

Test dataset											
	**N = 35** (F/M: 21/14)					**N = 36** (F/M: 23/13)					
	Mean ± Std	Range	Quartile 25%	Quartile 50%	Quartile 75%	Mean ± Std	Range	Quartile 25%	Quartile 50%	Quartile 75%	P-Value
Age	68.51 ± 7.12	56 – 79	63	69	74	66.11 ± 6.53	56–78	61	66	70	0.16
MMSE score	18.86 ± 5.8	5 – 27	16	20	24	28.91 ± 1.25	24–30	28	29	30	0.00
Audio length	79.42 ± 36.80	28.39 – 150.15	51.52	70.20	106.97	66.35 ± 28.58	22.35–135.68	44.4	66.04	77.69	0.17
Speech rate	91.18 ± 34.47	26.83 – 181.3	70.4	93.39	111.41	108.46 ± 33.97	48.28–201.95	87.34	104.86	132.54	0.04
Pause ratio	0.51 ± 0.17	0.17 – 0.84	0.44	0.49	0.63	0.42 ± 0.16	0.18–0.90	0.30	0.40	0.48	0.008

**Table 3:** Participants Characteristics of PREPARE Phase 2 dataset (“PREPARE dataset”)

Development (Training) dataset											
Attributes	Cognitively impaired participants (CI)					Cognitively normal participants (CN)					
	N = 456 (F/M = 255/201)					N = 608 (F/M = 348/260)					
	Mean ± Std	Range	Quartile 25%	Quartile 50%	Quartile 75%	Mean ± Std	Range	Quartile 25%	Quartile 50%	Quartile 75%	P-Value
Age	75 ± 8.25	47–97	69.75	77	81	74.88 ± 8.33	46–96	70	80	81	0.55
Audio length	26.8 ± 5.54	5.36–30	24.78	30	30	26.77 ± 5.4	6.3–30	24.22	30	30	0.02
Speech rate	133 ± 44.53	42.5–203	110.27	142.29	166.49	151 ± 31.9	91.57–203.8	130.8	156	175.5	6.4e-9
Pause ratio	0.14 ± 0.14	0.01–0.67	0.03	0.10	0.20	0.1 ± 0.1	0.01–0.44	0.02	0.07	0.16	3e-4

Test Dataset											
	**N = 115** (F/M: 64/51)					**N = 152** (F/M: 87/65)					
	Mean ± Std	Range	Quartile 25%	Quartile 50%	Quartile 75%	Mean ± Std	Range	Quartile 25%	Quartile 50%	Quartile 75%	P-Value
Age	74.9 ± 9	49–94	67	77	81	74.42 ± 8.77	48–90	69	79	81	0.85
Audio length	26.8 ± 5.61	1.11–30	24.84	30	30	27 ± 5.28	7.1–30	25.34	30	30	0.21
Speech rate	144.30 ± 40.85	64–205.77	122.47	153.67	169.27	158 ± 28.1	116.34–214.93	137.56	156.72	181.24	0.07
Pause ratio	0.11 ± 0.11	0.0–0.51	0.02	0.09	0.14	0.1 ± 0.1	0.01–0.36	0.02	0.07	0.14	0.79

#### Pitt corpus

The DementiaBank [102] Pitt corpus (hereafter referred to as the Pitt corpus) dataset includes 237 participants, divided by the publisher into a training set of 166 participants and a test set of 71 participants. All participants underwent an extensive neuropsychiatric evaluation, including medical history, physical and neurological exams, a semi-structured psychiatric interview, and neuropsychological assessment, and were reviewed by the University of Pittsburgh Alzheimer Disease Research Center consensus panel for diagnosis (details provided in Appendix B). Table 2 summarizes the cohort, comprising cognitively impaired (CI, case group) and cognitively normal (CN, control group) individuals. All participants were older than 53, with women representing more than 60% of each group. Mini-Mental State Examination [103] (MMSE) scores, which has a range from 0 to 30, varied from 3 to 28 in the CI group, reflecting mild to severe cognitive impairment, while scores in the CN group were all above 24, indicating normal cognition. Significant differences in speech rate, audio length, and pause ratio between the CI and CN groups were assessed using the Mann–Whitney U test [104]. CI participants exhibited a lower speech rate, longer audio length, and higher pause ratio compared to CN participants. We used Mann–Whitney U test to compute p-values, as it does not require assumptions of normal distribution or homogeneity of variances [104].

#### PREPARE dataset (NIA challenge, Phase 2)

Table 3 presents the PREPARE Phase 2 dataset (hereafter referred to as the “PREPARE dataset”), released by the National Institute on Aging (NIA) as part of the PREPARE Challenge to support development of voice-based tools for early detection of cognitive impairment. The dataset comprises brief (≤ 30-s), English-language speech recordings collected from older adults across four distinct speech-production tasks: (1) Cookie-Theft picture description, (2) story (Craft) recall, including immediate and delayed recall, (3) semantic fluency tasks, and (4) interactive dialogue with Amazon Alexa. These tasks capture a range of cognitive-linguistic functions, including spontaneous speech generation, lexical retrieval, memory, and conversational dynamics. The development (training) set includes 1,064 participants, including 456 cognitively impaired (CI) and 608 cognitively normal (CN) individuals, while the held-out test set includes 267 participants, including 115 CI and 152 CN individuals. Participants across both sets had a mean age of approximately 75 years.

In the development set, CI participants exhibited significantly slower speech (133 ± 44.53 words per minute) than CN participants (151 ± 31.9 words per minute, p = 6.4 × 10⁻9). CI participants also showed a higher pause ratio (0.14 ± 0.14 vs. 0.10 ± 0.10) and slightly longer audio duration. These patterns remained directionally consistent in the test set, where CI participants again showed slower speech, more frequent pauses, and longer recordings, although not all differences reached statistical significance. Overall, the PREPARE dataset provides a large, task-diverse, English-language benchmark for evaluating the generalizability and robustness of speech-based cognitive screening models such as SpeechDETECT.

Because PREPARE is a newly released dataset without established published baselines for structured acoustic pipelines, we constructed matched comparison baselines by applying six widely used acoustic feature toolkits under the same preprocessing, feature-selection, classifier-training, and evaluation framework used for SpeechDETECT. This design ensures that comparisons on PREPARE reflect differences in feature representation rather than differences in downstream modeling or evaluation procedures.

### Baseline feature sets

To establish matched baselines for both datasets, including the newly introduced PREPARE corpus, we applied the same downstream classification and evaluation pipeline to features derived from six established acoustic toolkits frequently used in speech-based cognitive impairment studies: AVEC 2013, ComParE 2013/2016, GeMAPS/eGeMAPS, IS10-Paralinguistic, Emo-Base Large, and COVAREP. For both datasets, each toolkit was processed using the same normalization, feature-selection, classifier-training, and evaluation procedures as SpeechDETECT, allowing direct performance comparison under matched conditions.

### Informative features representation

We used SHAP [105] (SHapley Additive exPlanations) to interpret how each feature influences model predictions. SHAP values quantify both the magnitude and direction of each feature’s impact, allowing for a nuanced understanding of feature importance. This helps in determining which features most significantly contribute to the model’s performance.

### Analysis of misclassifications

To analyze misclassifications—false positives (FP, labeled as CI) and false negatives (FN, labeled as CN)—we identified the most influential features from each of the eight domains (eight features total) based on their SHAP values. We then applied the non-parametric Mann–Whitney U test [104] to compare these features across FP vs. TP (true positives, labeled as CI), FP vs. TN (true negatives, labeled as CN), FN vs. TN, and FN vs. TP. The U test was chosen for its minimal assumptions regarding data distribution and suitability for small samples. It yields an Effect Size and a P-value, where the Effect Size provides insight into both the magnitude and direction of differences between groups. For the P-value, we set the threshold at 0.1 to allow a less restrictive criterion for identifying potential differences [104].

#### SHAP and error analysis scope

We restricted detailed SHAP and error analyses to the Pitt corpus because its single, well-controlled Cookie-Theft task provides relatively long, continuous speech samples (≈60–90 s), enabling more stable frame-level attribution and clearer interpretation of acoustic patterns associated with cognitive status. In contrast, the PREPARE Phase 2 corpus comprises shorter recordings (≤ 30 s) across four cognitively distinct tasks—picture description, immediate and delayed story recall, semantic fluency, and Alexa dialogue—each eliciting different speech characteristics. Pooling these tasks for SHAP analysis would confound task-specific effects with clinical effects, while task-stratified SHAP analyses would yield heterogeneous attribution profiles that are not directly comparable. For these reasons, we limit SHAP and error analyses to the more controlled and task-homogeneous Pitt corpus.

## Results

**Fig. 4:**
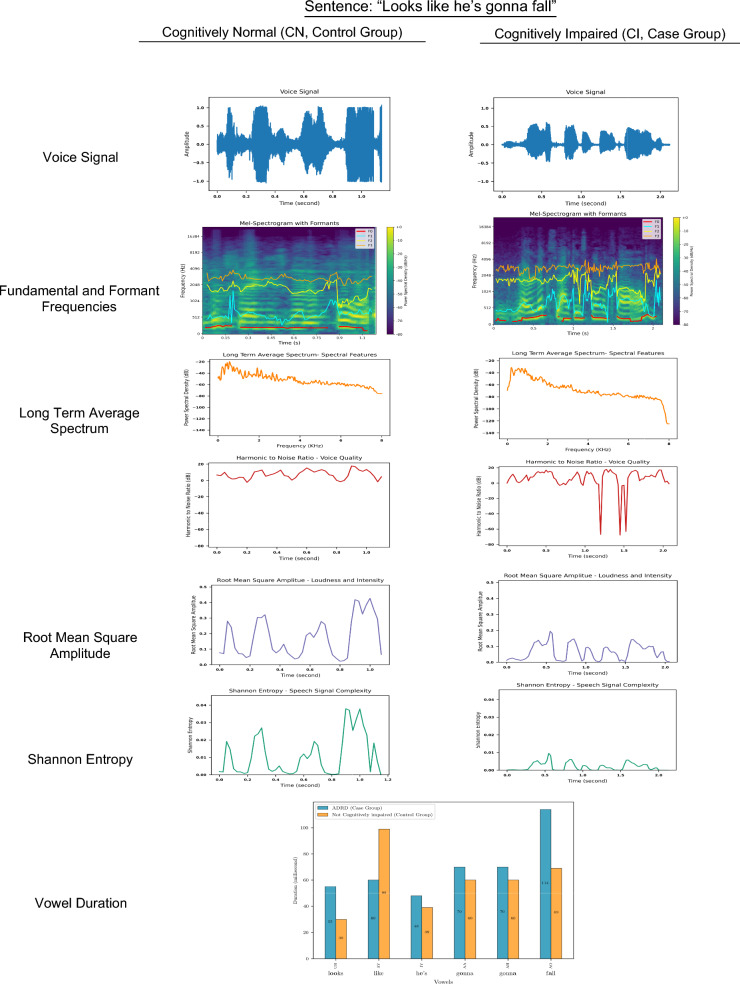
Visualization of acoustic and temporal features of sentence “**Looks like he’s gonna fall”** spoken by two subjects, one from the CI group and one from the CN group

The pipeline’s visualization component enables the depiction of acoustic and temporal features across eight analytical domains. Figure 4 illustrates the voice signal, key acoustic features indicative of vocal traits, and vowel duration as a temporal feature for two subjects (one from the CI group and one from the CN group) speaking the sentence, "Looks like he’s gonna fall." The analysis for “Fundamental and Formant Frequencies” shows greater fluctuation in fundamental and formant frequencies for the CI subject compared to the CN subject, suggesting challenges in controlling speech production organs. Additionally, the “Root Mean Square Amplitude” indicates that the CI subject’s speech has lower energy levels than the CN subject. As a temporal feature, “Vowel Durations” are generally longer for the CI subject, reflecting slower speech dynamics.

### Classification performance of SpeechDETECT across two datasets Pitt corpus and PREPARE dataset

**Fig. 5:**
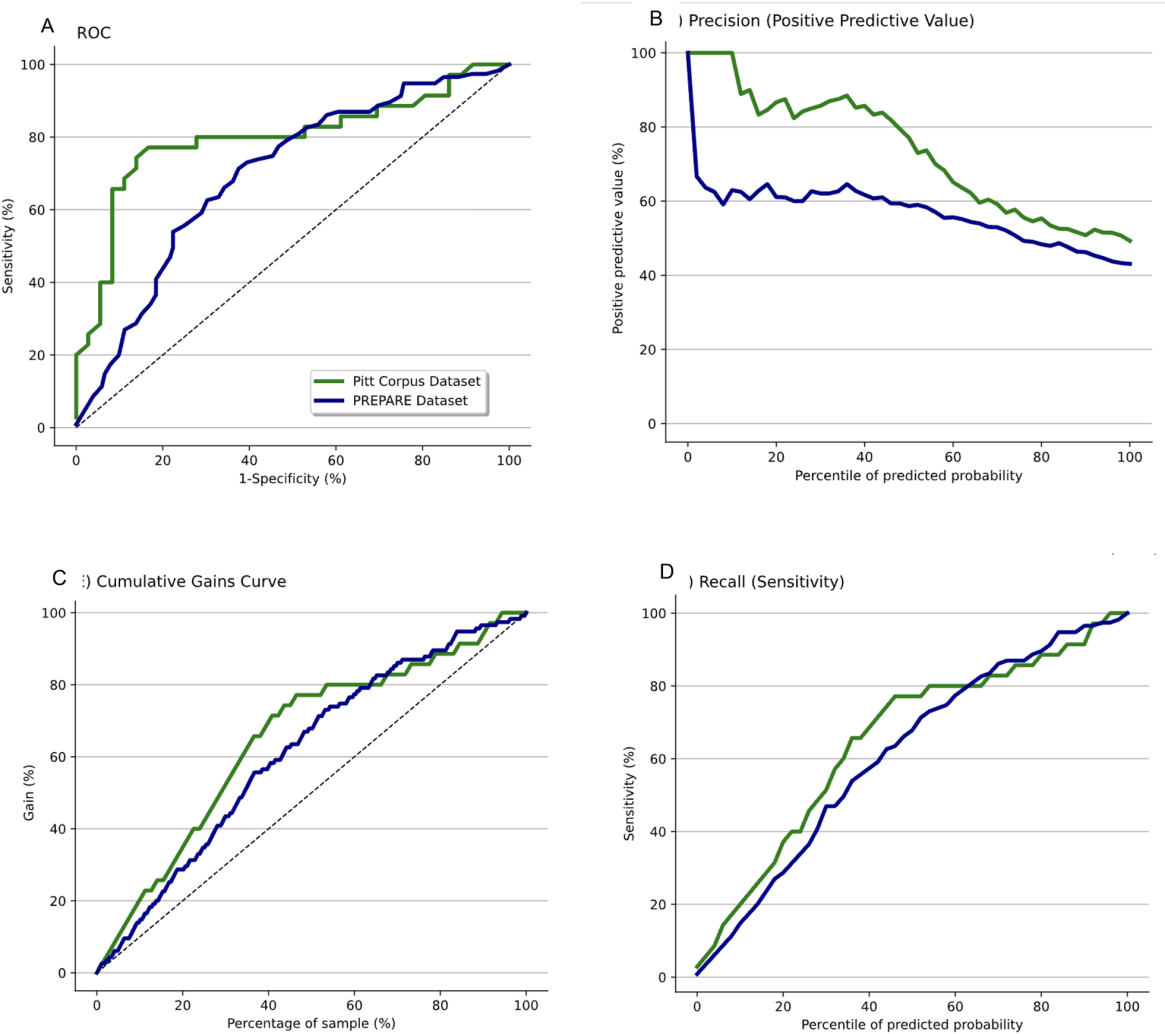
Performance of SpeechDETECT on two held-out test sets—the DementiaBank Pitt Corpus (green) and the PREPARE Phase 2 dataset (blue). **A** Receiver-operating-characteristic (ROC) curves highlight overall discrimination (AUC = 0.80 for Pitt corpus; 0.70 for PREPARE dataset). **B** Precision (positive-predictive value) plotted against percentiles of predicted probability shows that precision stays above 80% in the Pitt corpus and about 65% in PREPARE dataset within the top 40% of ranked predictions**. C** Recall (sensitivity) curves indicate that the model detects > 80% of cognitively-impaired participants above the 60th percentile in Pitt corpus and > 70% in PREPARE dataset**. D** Cumulative-gains curves demonstrate that selecting the top 40% of participants by predicted probability captures ~ 70% of CI cases in Pitt and ~ 63% in PREPARE dataset

The MLP classifier—configured with a single 350-unit hidden layer and a learning rate of 1 × 10^–3^ performed best when trained on PCA-transformed SpeechDETECT features. On the DementiaBank Pitt test set the model achieved an F1-score of 0.81 and an AUC-ROC of 0.80. On the more heterogeneous PREPARE Phase 2 test set (shorter, task-diverse recordings), it obtained an F1-score of 0.67 and an AUC-ROC of 0.70 (Fig. 5A).

The precision (positive-predictive-value) curve in Fig. 5B shows that precision stays above 80% for the top 40% of ranked predictions in the Pitt corpus, while remaining around 65% for PREPARE.

The recall (sensitivity) curve in Fig. 5C indicates that the model detects > 80% of cognitively-impaired (CI) participants above the 60th percentile in Pitt and > 70% in PREPARE.

Finally, the cumulative-gains curve in Fig. 5D demonstrates that selecting the top 40% of participants by predicted probability captures ≈ 70% of CI cases in Pitt and ≈ 63% in PREPARE.

Taken together, these curves confirm that SpeechDETECT offers strong discrimination on the structured, single-task Pitt corpus and maintains clinically useful precision and sensitivity on the shorter, multi-task PREPARE recordings despite their greater variability.

### Performance comparison of SpeechDETECT with established acoustic toolkits

**Fig. 6:**
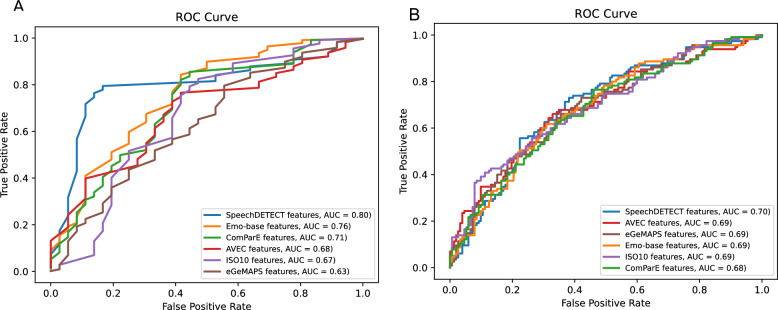
**A** ROC curves for SpeechDETECT and six established acoustic toolkits on the DementiaBank test set. SpeechDETECT achieved the highest AUC (0.80), outperforming Emo-Base Large (0.76), ComParE (0.71), and other widely used toolkits**.B** ROC curves for SpeechDETECT and six established acoustic toolkits on the PREPARE Phase 2 test set. Although the AUC differences were smaller, SpeechDETECT maintained the highest AUC (0.70) among all feature sets

Figure 6 presents a side-by-side comparison of SpeechDETECT and six established acoustic feature sets evaluated using the same classification pipeline described in Sect. "Baseline Feature Sets". On the Pitt corpus test set **(**Fig. 6.A), SpeechDETECT achieved the highest AUC (0.80), outperforming all other toolkits, including Emo-Base Large (AUC = 0.76), ComParE (0.71), and eGeMAPS (0.63). On the test set of PREPARE dataset (Fig.  6.B), which contains shorter recordings across multiple task types, SpeechDETECT again achieved the highest AUC (0.70), though the range of AUCs across toolkits was narrower (0.68–0.70). These results indicate that while all toolkits perform similarly on PREPARE, SpeechDETECT demonstrates stronger discriminative performance on the more structured and homogeneous DementiaBank dataset, and retains a slight edge under varied recording conditions.

### SHAP values visualization (on Pitt corpus)

**Fig. 7:**
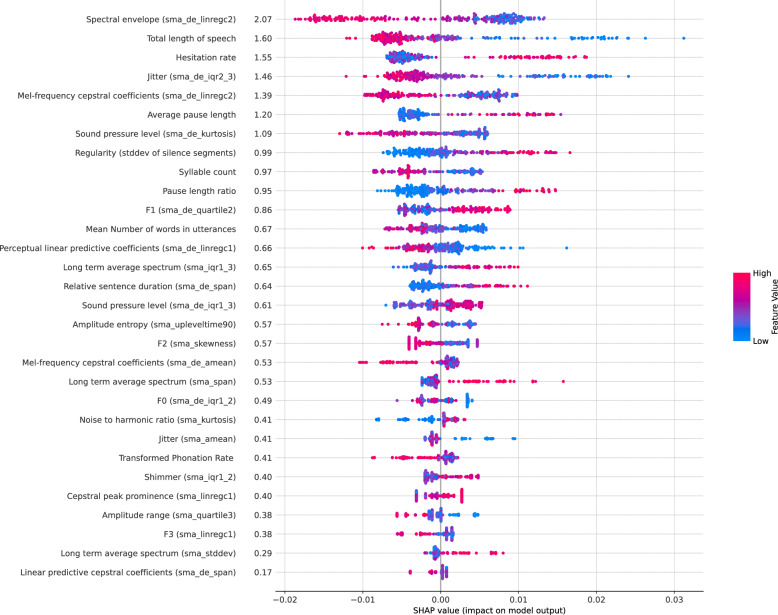
SHAP [116] summary plot for top 30 acoustic features. Each point on the plot represents a subject, with the color indicating the feature value for that subject (red for high and blue for low). Positive SHAP values (X-axis) indicate that the feature contributes to pushing the pipeline toward predicting CI, while negative values indicate that the feature pushes the model toward predicting CN. The further a feature’s value is from the vertical line at X = 0, its influence is greater on the pipeline’s prediction outcome. The values next to each feature represent the corresponding sum of the absolute SHAP values for that feature across all subjects

**Fig. 8:**
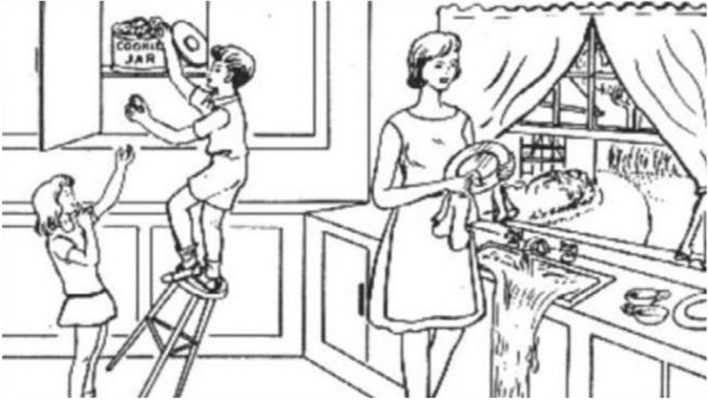
Cookie-theft speech description task. Source: Figueiredo, S., & Barfod, V. (2012). Boston Diagnostic Aphasia Examination (BDAE) Chicago [117]

Figure 7 presents the SHAP summary plot for the 30 most influential Speech-DETECT features. To make the directional patterns explicit, Table 4 groups those features by whether higher, lower, or task-dependent values push the classifier toward the CI prediction and maps each one to the acoustic domain it represents.

**Table 4:** Direction of influence and domain of the top SpeechDETECT features contributing to CI prediction based on SHAP analysis (Pitt corpus)

	Features	SpeechDETECT domain	Concise clinical interpretation
Higher Value ⟶ CI	Hesitation rate	Speech fluency	More fillers/pauses → lexical retrieval difficulty
	Average pause length	Speech fluency	Longer silences between phrases
	Pause-length ratio	Speech fluency	Greater proportion of silence
	Regularity (stdev of silence)	Rhythmic structure	Uneven pause timing
	LTAS (sma_iqr1_3)	Spectral shape	Flatter long-term spectrum
	Spectral envelope (sma_de_linregc2)*	Cepstral/Spectral	Reduced spectral tilt
	MFCC (sma_de_linregc2)*	Cepstral/Spectral	Less complex spectral contour
	PLP coeff. (sma_de_linregc1)	Cepstral/Spectral	Spectral flattening
	Sound-pressure level Δ IQR	Loudness/Intensity	Narrow dynamic range
	Amplitude entropy	Signal complexity	Lower acoustic variability
	NHR (sma_kurtosis)	Voice quality	More breathiness/noise
	Shimmer (Δ IQR₁–₂)	Voice quality	Unstable amplitude
	Transformed phonation rate	Rhythmic structure	Slower syllabic articulation
	Relative sentence duration	Production dynamics	Slow overall delivery
Lower Value → CI	Total length of speech	Production dynamics	Shorter narratives
	Syllable count	Production dynamics	Fewer syllables produced
	Mean # words / utterance	Production dynamics	Simpler sentence structure
	Jitter (Δ IQR₂–₃)	Voice quality	Reduced pitch micro-variation
	F0 (Δ IQR₂)	Frequency parameters	Narrow pitch range
	F1 (sma_de_quartile2)	Frequency parameters	Centralised vowel space
	F2 (sma_skewness)	Frequency parameters	Reduced vowel distinctiveness
	F3 (sma_linregc1)	Frequency parameters	Blurred high-formant articulation
	Sound-pressure level (kurtosis)	Loudness/Intensity	Flatter intensity contour
	Spectral centroid Δ span	Cepstral/Spectral	Lower spectral “brightness”
	LTAS (sma_stddev)	Spectral shape	Monotonous spectrum
Mixed (task-dependent)	Spectral envelope Δ span	Cepstral/Spectral	Task-specific spectral changes
	MFCC (sma_de_mean)	Cepstral/Spectral	Variable mel-scale shifts
	Amplitude range (Δ quartile3)	Loudness/Intensity	Speaker-dependent loudness spread
	Cepstral peak prominence	Cepstral/Spectral	Varies with phonatory effort

* sma_de_linregc2/linregc1 indicate the slope of a linear regression through the delta contour; larger positive slopes correspond to flatter or less dynamic spectral shapes

These results show that several speech features reliably distinguish cognitively impaired from cognitively normal participants. Increased hesitation rate, pause duration, and pause ratio reflect impaired fluency and lexical retrieval in CI. Indicators of spectral flattening—including MFCC slope, LTAS, and PLP coefficients—suggest reduced articulatory variability. Elevated jitter, shimmer, and NHR point to unstable vocal fold vibration and increased breathiness. Additionally, reduced speech length, syllable count, and mean utterance length indicate simpler and shorter output, consistent with cognitive load effects. Together, these patterns confirm that SpeechDETECT captures clinically relevant acoustic changes associated with early cognitive impairment. For the rationale behind omitting SHAP results for the PREPARE dataset, please see the “SHAP and Error-Analysis Scope” subsection of the Methodology section.

### Analysis of misclassifications (on DementiaBank)

Table 5 presents the distribution of gender, MMSE scores, and the most important acoustic features across eight domains for the classification groups (TP, FN, TN, FP; see “Analysis of Misclassification” section). Table 6 summarizes the U test results, including effect sizes and p-values, for statistical comparisons among these groups.

**Table 5:** Confusion matrix of the SpeechDETECT performance on the test dataset

	True label		
		CI (cognitively impaired)	CN (cognitively normal)
Predicted label	CI	**TP: 28** Gender (M/F): 12/16	**FP: 6** Gender (M/F): 3/3
		**Variable (mean ± std)** MMSE: 18.32 ± 6.22 Total length of speech: 0.46 ± 0.17 Hesitation rate: 0.06 ± 0.03 Jitter: 0.06 ± 0.02 MFCC: -0.35 ± 0.86 SPL: 5.39 ± 2.76 Amplitude Entropy: 23.59 ± 7.42 NHR: 640.31 ± 524.03 Transformed phonation rate: 0.62 ± 0.08	**Variable (mean ± std)** MMSE: 29.0 ± 0.89 Total length of speech: 0.54 ± 0.14 Hesitation rate: 0.04 ± 0.02 Jitter: 0.06 ± 0.02 MFCC: 0.23 ± 0.44 SPL: 4.79 ± 3.86 Amplitude Entropy: 23.56 ± 5.18 NHR: 510.85 ± 376.0 Transformed phonation rate: 0.62 ± 0.11
	CN	**FN: 6** Gender(M/F): 2/4	**TN: 30** Gender (M/F): 11/19
		**Variable (mean ± std)** MMSE: 20.5 ± 3.15 Total length of speech: 0.60 ± 0.15 Hesitation rate: 0.04 ± 0.02 Jitter: 0.08 ± 0.01 MFCC: 1.26 ± 0.83 SPL: 5.72 ± 1.73 Amplitude Entropy: 25.24 ± 9.72 NHR: 345.08 ± 247.67 Transformed phonation rate: 0.77 ± 0.08	**Variable (mean ± std)** MMSE: 28.83 ± 1.34 Total length of speech: 0.59 ± 0.16 Hesitation rate: 0.04 ± 0.02 Jitter: 0.07 ± 0.02 MFCC: 0.59 ± 1.50 SPL: 7.42 ± 2.43 Amplitude Entropy: 28.55 ± 6.81 NHR: 399.04 ± 263.44 Transformed phonation rate: 0.75 ± 0.10

**Table 6:** Effect size and p-values of the eight most informative acoustic and temporal features (the most informative from each domain) presented in Table 5

variable	TP-FN		TN-FN		TN-FP		TP-FP	
	P-value	Effect size	P-value	Effect size	P-value	Effect size	P-value	Effect size
MMSE	0.51	0.18	1e-4	−0.99	1	0	3e-4	0.94
Total length of speech	0.13	0.40	0.82	−0.07	0.22	−0.33	0.34	0.26
Hesitation rate	0.25	-0.30	0.40	0.22	0.92	0.03	0.15	-0.39
Jitter	0.01	0.64	0.20	0.34	0.52	−0.18	0.61	0.14
MFCC	0.0001	0.88	0.06	0.49	0.95	−0.02	0.10	0.44
SPL	0.37	0.25	0.12	−0.41	0.02	−0.62	0.13	-0.40
Amplitude Entropy	0.71	0.10	0.49	−0.19	0.09	−0.44	0.98	0.01
NHR	0.34	-0.26	0.79	−0.07	0.54	0.17	0.89	-0.07
Transformed phonation rate	0.001	0.83	0.73	0.1	0.009	−0.69	0.59	0.15

The U test revealed significant differences in three acoustic features between the TP and FN groups: Jitter (p = 0.01), MFCC (p = 0.0001), and Transformed Phonation Rate (p = 0.001), all meeting the significance criterion of p ≤ 0.1 (see Table 6). No significant differences were observed for the remaining five features (p > 0.1). Comparisons between the FN and TN groups across all eight features showed no significant differences, suggesting similar speech patterns between the CI group (FN group, six subjects) and the CN group (TN group). However, MMSE comparisons indicated notable cognitive disparities between these groups.

Further analysis showed significant differences between the FP and TN groups in Sound Pressure Level (p = 0.02), Amplitude Entropy (p = 0.09), and Transformed Phonation Rate (p = 0.009), also meeting the p ≤ 0.1 threshold (see Table 6). No other features differed significantly (p > 0.1). Comparisons between the FP and TP groups revealed broadly similar acoustic profiles, implying that the FP group’s speech patterns resembled those of cognitive impairment. Nonetheless, MMSE scores exposed substantial cognitive differences, highlighting a disconnect between speech features and cognitive status.

Overall, these findings suggest that subjects misclassified in both the CI and CN categories exhibited deviations in specific acoustic features that did not align with their clinically expected diagnostic profiles.

the rationale behind omitting error analysis for the PREPARE dataset, please see the “SHAP and Error-Analysis Scope” subsection of the Methodology section.

## Discussion

This study presents SpeechDETECT, an explainable and validated speech-processing pipeline that characterizes vocal traits and temporal aspects of speech across eight acoustic domains: frequency, cepstral coefficients and spectral features, voice quality, loudness, intensity, speech signal complexity, speech fluency, rhythmic structure, and speech production dynamics. A multilayer perceptron model, trained on the DementiaBank dataset—a widely used benchmark for cognitive impairment detection—achieved an AUC-ROC of 0.80 and an F1-score of 0.81, outperforming prior studies that analyzed acoustic features in this dataset.

Importantly, SpeechDETECT also generalized to the task-diverse PREPARE corpus. Despite recordings being ≤ 30 s and spanning four distinct speech tasks, the same MLP model achieved an AUC-ROC of 0.70 and an F1-score of ≈ 0.67***.*** Cumulative-gains analysis showed that screening the top 40% of ranked participants captured ≈ 63% of cognitively-impaired cases. These results indicate that SpeechDETECT retains clinically useful sensitivity and precision even when speech is brief, heterogeneous, and collected in less-controlled environments—an essential property for real-world deployment in primary-care and remote-monitoring settings.

A key strength of SpeechDETECT is its use of SHAP-based feature attribution, which quantifies and visualizes the contribution of individual acoustic features to classification outcomes. SHAP analysis identified speech fluency, rhythmic structure, frequency parameters, and cepstral coefficients as the strongest predictors of cognitive impairment, reinforcing prior findings. Notably, speech production dynamics and speech signal complexity—particularly total speech length and amplitude entropy—also emerged as important yet less explored markers of cognitive decline in previous studies.

Our error analysis provided additional insights into early cognitive impairment detection. Among false-negative (FN) cases, key acoustic features resembled those of the incorrectly assigned diagnostic group, suggesting that some early-stage patients with cognitive impairment may retain certain speech characteristics despite underlying pathology. Conversely, in false positive cases, healthy individuals displayed acoustic features resembling those of cognitively impaired patients, indicating potential overlap or confounding factors in the speech markers. These findings highlight the need for multimodal screening approaches that integrate speech with clinical evidence and biomarkers (e.g., blood tests, MRI data) to enhance diagnostic accuracy.

With the emergence of disease-modifying monoclonal antibody therapies for Alzheimer’s disease, early detection is more critical than ever. Speech-based screening offers a scalable, non-invasive tool for identifying at-risk individuals, particularly in primary care and home healthcare settings, [106] where access to specialized cognitive assessments is often limited. While prior studies have explored EHR-based screening models, they have difficulties with low sensitivity, particularly in early-stage cognitive impairment [107–109]. Additionally, widely used cognitive assessment tests such as the MMSE often lack sensitivity for early-stage detection. Notably, one participant in the DementiaBank dataset had a perfect MMSE score of 30 [110] yet was clinically diagnosed with cognitive impairment— an outcome captured by SpeechDETECT acoustic and temporal analysis, demonstrating the strong potential of speech as an early biomarker.

Taken together, these findings indicate that SpeechDETECT provides an integrated and explainable framework for speech-based detection of cognitive impairment across two datasets. By combining domain-informed acoustic analysis with interpretable modeling, the pipeline supports classification performance while also providing clearer insight into the speech characteristics contributing to model decisions. These properties may enhance its relevance for real-world clinical use, where transparency, robustness, and interpretability are important for adoption. More broadly, SpeechDETECT may also be relevant for assistive and remote screening settings, including home-based monitoring and telehealth, where brief and interpretable speech assessments may support earlier identification of cognitive changes. Future work should evaluate the pipeline in more diverse populations and under real-world deployment conditions.

Despite these promising findings, clinical adoption of speech-based tools remains limited [111–113]. Logistical challenges, clinician hesitancy, and a lack of standardized workflow integration continue to hinder widespread implementation. [114] Future research should focus on identifying barriers and facilitators to clinical uptake, ensuring that speech processing tools are developed with usability and scalability in mind. Policy-level support is also needed to advance speech-based screening as a routine component of cognitive health assessments.

### Limitations

Our study focused on acoustic markers of cognitive impairment and did not incorporate pre-trained transformer models, which could further enhance detection by capturing complex linguistic and acoustic patterns [14, 115]. Due to the substantial data requirements of transformer-based models, we plan to fine-tune SpeechDETECT on a larger, racially diverse dataset currently being collected at Columbia Medical Center and a home healthcare agency in New York City. Additionally, the DementiaBank dataset primarily includes White participants, limiting generalizability to diverse populations. Since racial and ethnic minorities face both higher dementia risks and significant healthcare disparities, future research should prioritize representative datasets to ensure equity in early detection strategies.

## Conclusion

We introduced SpeechDETECT, an explainable and integrated speech-processing pipeline that leverages acoustic biomarkers for early cognitive impairment detection. Our findings highlight the importance of speech as a sensitive, non-invasive marker of cognitive decline, offering a promising alternative to traditional screening tools. Future work should expand upon these insights by integrating linguistic features and refining speech-based detection methods for diverse populations, paving the way for more accessible and equitable cognitive health assessments.

## Data Availability

The dataset used for this study is from DementiaBank dataset, the largest publicly available benchmark with audio recordings for the picture-description task.

## Appendix

See Fig. 8 and Tables 7,8 and 9

## Appendix B

Inclusion and exclusion criteria for recruiting patients for the ADRD study [102] (DementiaBank Dataset Pitt Corpus).

### Recruitment

Individuals enrolled in the Alzheimer’s Disease Research Program (ARP) were mainly recruited from various sources, including The Benedum Geriatric Center at the University of Pittsburgh Medical Center and local medical professionals. To facilitate patient recruitment, the research team used public service announcements, and presentations at Alzheimer’s organizations and support groups.

### Exclusion criteria

Individuals were excluded if they had severe behavioral or psychiatric symptoms, significant speech impairments, central nervous system diseases (e.g., brain tumors, seizure disorders, subdural hematomas, cranial arteritis), or required emergent care for conditions like uncontrolled pain or wound infections. Frequent use of high-dose opioid analgesics was also an exclusion criterion.

### Inclusion criteria

Individuals diagnosed with ADRD and exhibiting related symptoms were eligible for the case group, while those with no history of cognitive impairment were eligible for the control group. Individuals with initial interest underwent an extensive neuropsychiatric evaluation, including medical history, physical and neurological exams, a semi-structured psychiatric interview, and neuropsychological assessment. A psychiatric nurse assessed physical and cognitive limitations and caregiving burden. The individuals also completed laboratory tests, including hematology, blood chemistry, liver and thyroid function tests, vitamin levels, EEG, CT scan of the head, and a chest X-ray. Neuropsychological data were not used as selection criteria. To qualify as a "patient," individuals needed to show a history of progressive cognitive and functional decline and an abnormal mental status examination. Medical and psychiatric evaluations were designed to rule out conditions that could independently cause dementia.

**Table 7:** Complete list of features

Frequency parameters
**F0/Pitch** is the lowest sound wave frequency, set by vocal fold vibrations, and defines perceived pitch in human voice [66]
**Jitter** is the variation in the frequency or pitch of consecutive vocal cycles in speech, indicating the stability of vocal production [66]
**F1** is the first formant frequency, influenced by vertical position of the tongue within the oral cavity, critical in determining vowel quality [118]
**F2** is the second formant frequency, influenced by horizontal position of the tongue, critical for vowel sound differentiation. [118]
**F3 and above**: Higher formants like F3 relate more to fine articulatory details, including tongue and lip positioning, and are more challenging to articulate precisely when motor control or cognitive planning is impaired [118]
**Cepstral Coefficients and Spectral Features**
**Modulation Spectra Coefficients (MSC)** are derived from the modulation spectrum to capture the temporal dynamics and variations in energy across different frequency bands of a speech signal over time [119]
**Spectral centroid (CENTROIDS)** indicates the center of mass of the spectrum, reflecting the perceived brightness or sharpness of the sound [120]
**Long Term Average Spectrum (LTAS)** provides an average frequency spectrum over time, offering insights into energy distribution across frequencies and aiding in the analysis of speech patterns and vocal health**.** [63]
**Log Mel Spectrogram (LOG_MEL_SPECTROGRAM)** converts audio frequencies to the Mel scale and applies logarithmic scaling, matching human hearing perception. [121]
**Mel-frequency Cepstral Coefficients (MFCC)** capture an audio signal’s spectral characteristics on the mel scale, mirroring human ear perception [121]
**Linear Predictive Coefficients (LPC)** estimate the short-term spectral envelope of a speech signal by modeling each sample as a linear combination of previous samples [122]
**Linear Predictive Cepstral Coefficients (LPCC)** are derived from LPC, provide a more compressed and efficient representation of the LPC parameters (i.e. LPCCs capture the spectral shape of speech with fewer cepstral coefficients) [123]
**Spectral Envelope (ENVELOPE)** represents the smooth curve outlining the peaks of a sound spectrum, capturing the overall shape and distribution of energy across frequencies [124]
**Cepstral Peak Prominence (CPP)** quantifies the amplitude difference between the cepstrum peak (representing voice harmonics) and the surrounding baseline level in the cepstral domain. Higher CPP indicates a more defined harmonic structure and lower CPP indicates a breathy or disordered voice quality, that can be associated with issues in vocal fold vibrations or other phonatory functions [125]
**Perceptual Linear Predictive Coefficients (PLP)** simulates the vocal tract as series of digital filters (representing formant frequencies), and predicts given speech by linear combination of past speech samples [126]
**Line Spectral Pairs (LSP)** represents linear prediction coefficients (LPC) for transmission over a channel, providing a straightforward view of spectral content and aiding in the analysis of harmonic and formant structures [127]
**Voice Quality**
**Amplitude Perturbation Quotient (APQ2)** quantifies the variation in amplitude of the vocal waveform over time, offering insights into voice quality dynamics [128]
**Shimmer (SHIMMER)** is the variation in amplitude or loudness between consecutive vocal cycles in speech, reflecting voice stability and quality [66]
**Harmonic-to-Noise Ratio (HNR)** quantifies the ratio of harmonic (periodic) sound to noise (aperiodic) sound in a voice, reflecting its timbral smoothness and quality [129]
**Alpha Ratio (ALPHA RATIO)** is the ratio of the summed energy from 0–1000 Hz and 1–5 kHz.[130]
**Hammarberg Index (HAMMARBERG_INDEX)** calculates the ratio of energy in the high-frequency region to the main spectral peak, with higher values typically indicating a brighter or more strained voice quality [131]
**Harmonicity (HARMONICITY)** measures how sound frequencies align with the fundamental frequency, influencing pitch and voice quality [132]
**Loudness and intensity of the sound**
**Amplitude Range** refers to the difference between the maximum and minimum amplitude levels in a sound wave [133]
**Root Mean Square (RMS) amplitude** represents the square root of the average of the squares of the amplitude values in a sound wave, indicative of the wave’s overall energy or power [133]
**Sound Pressure Level (SPL)** measures the intensity or loudness of sound in decibels (dB), based on its pressure variations relative to 20 micropascals, the lowest hearing threshold in air related to human hearing [134]
**Peak amplitude (PEAK)** refers to the maximum level of signal strength or intensity in a sound wave, representing the highest point of the wave’s fluctuation
**Short time energy (STE)** refers to the measure of the average power or intensity of a sound signal over a brief, specified time period [135]
**Intensity (INTENSITY)** represents the cube root of the normalized average of the squares of the amplitude values in a sound wave, serving as an indicator of the wave’s overall energy or power [136]
**Speech Signal Complexity**
**Higuchi Fractal dimension (HFD)** quantifies the complexity or irregularity of a waveform by analyzing its fractal characteristics [76]
**Shannon entropy**–**Frequency (FREQ_ENTROPY)** measures how spread out or concentrated the energy distribution is across the frequency spectrum, capturing the unpredictability or complexity in a signal’s frequency content [75]
**Shannon entropy**–**Amplitude (AMP_ ENTROPY)** quantifies the randomness or uniformity in a signal’s amplitude values, reflecting how evenly (or unevenly) the amplitudes are distributed [75]
**Zero-Crossing Rate (ZCR)** measures how frequently the waveform crosses zero amplitude, aiding in identifying the speech signal’s oscillation and rhythm and pace in speech patterns [80, 137]
**Rhythmic structure**
**Pairwise Variability Index (PVI)** quantifies the rhythmic variation in speech by measuring the timing differences between successive vocal elements [80] • PVI_0: a metric that measures the variation in speech rhythm by calculating the difference in duration between consecutive speech segment intervals and averaging those differences over an utterance • PVI_1: normalized PVI by the average length of speech segments
**Vowel Duration** (**VOWEL_DURATION**) measures the time vowels and consonants are articulated in speech, with particular emphasis on vowel duration. This focus is critical for detailed phonetic analysis, providing key insights into speech pronunciation and clarity [138]
**Voice segments metrics** assess the consistency and frequency of voiced part of speech by evaluating the temporal regularity and average number of voice segments per second, which is crucial for analyzing spoken language rhythm and flow.^79^ This includes the following metrics: • Average number of speech segments (AVERAGE_NUM_OF_SPEECH_SEGMENTS): The mean count of distinct utterances in audio recordings • Regularity (REGULARITY) measures the variation in speech rhythm by calculating the difference in duration between consecutive intervals (considers both speech and pause segments) and averaging those differences over an utterance
**Vocalic Interval (VOCALIC INTERVAL)** measures the percentage of vocalic intervals, which represents the ratio of vowel duration to total speech duration [139]
**Phonation rate metrics** quantifies vocal fold activity by calculating the percentage of phonation time and its arcsine-transformed rate, offering insights into the rhythm and dynamics of vocal expression [140].This includes the following features: • Percentage phonation time (PERCENTAGE_PHONATION_TIME) measures the proportion of total recording time during which a speaker is actively voicing • Transformed phonation rate (TRANSFORMED_PHONATION_RATE) represents a normalized measure of phonation occurrences, adjusted through specific transformations to provide insights into vocal patterns and speech dynamics • Phonation to Syllable (PHONATION_TO_SYLLABLE) quantifies the relationship between the duration of phonation and the number of syllables spoken, providing insights into speech fluidity and pacing
• Inter-syllabic pauses (MEAN_INTER_SYLLABIC_PAUSES) measures the mean duration of inter-syllabic pauses, providing insights into the pacing and rhythm of speech
**Token duration (TOKEN_DURATION)** measures the cumulative sum of durations of all tokens across all utterances, providing insights into the temporal characteristics of speech
**Verbal rate metrics** measure the dynamics of speech delivery, including the rate of syllables and words, articulation speed, and average utterance length, providing insights into speech rhythm [140] • Number of Words to Number of Pauses (NUM_WORDS_TO_NUM_PAUSES) measures the ratio of total words spoken to the number of pauses, indicating speech fluidity • Number of Syllables (NUM_OF_SYLLABLES) measures total count of syllables spoken in a given speech sample • Articulation Rate (ARTICULATION_RATE) measures the speed at which syllables are articulated, excluding pauses • Speech Rate by Syllable (SPEECH_RATE_SYLLABLE) measures the average number of syllables spoken per minute, including pauses • Speech Rate by Words (SPEECH_RATE_WORDS) measures the average number of words spoken per minute, including pauses
**Fluency of Speech**
**Voice Probability** quantifies the occurrences of disruptions in vocal continuity by counting the number of breaks during speech. This metric is crucial for assessing vocal stability, quality, and its impact on the fluency of speech [141]
**Count Pause segment (**COUNT_PAUSE_SEGMENTS) focuses on quantifying the frequency of pauses in speech by measuring the number of pause segments. This metric is key for assessing the structural dynamics of speech and interruptions in fluency [142]
**Pause length metrics** evaluate the duration of pauses in speech, by metrics such as the average and standard deviation of pause lengths, as well as the ratios of pause duration to both speech duration and total duration of the audio file [142] • Pause to Speech Segment Ratio (PAUSE_SPEECH_RATIO) measures the ratio of the number of pause segments to the number of speech segments, indicating the frequency of pauses relative to speech • Pause to Total Length Ratio (PAUSE_TOTALLENGTH_RATIO) measures the ratio of the total length of all pauses to the total length of the audio, reflecting the proportion of silence • Pause to Speech Duration Ratio (PAUSE_SPEECH_DURATION_RATIO) measures the ratio of the total length of pauses to the length of speech segments, indicating how much of the speech consists of pauses • Average Pause Length (PAUSE_LENGTHS_AVG) measures the average duration of pauses within a speech sample • Pause Length (PAUSE_LENGTH) measures the duration of a specific pause in the speech
**Pause and Hesitation rate metrics** quantify the frequency of speech interruptions by measuring hesitation rates, pauses per syllable and per word, and the ratio of pause segments to speech segments [78] • Hesitation Rate (HESITATION_RATE) measures the ratio of transcribed filler words to the total number of words, excluding filler words • Pause to Syllable Ratio (PAUSE_TO_SYLLABLE) measures the Ratio of the number of pause segments (excluding filler words) to the total number of syllables, indicating the frequency of pauses relative to speech complexity [78, 83, 142] • Pause to Tokens Ratio (PAUSE_TO_TOKENS) measures the ratio of the number of pause segments (excluding filler words) to the total number of tokens as determined by the NLTK package, reflecting pause frequency relative to speech units [78, 83, 142]
**Syllable related metrics** evaluate the structure of speech by quantifying the total syllable count, the number of syllabic intervals, and the duration of these intervals. These metrics are crucial for analyzing the patterns of language delivery and fluency [143] • Syllable Count (SYLLABLE_COUNT) measures the total number of syllables spoken within a given speech sample, used to assess speech complexity and fluency • Syllabic Interval Duration (SYLLABIC_INTERVAL_DURATION) measures the duration of intervals between syllables, providing insights into the timing and rhythm of speech delivery
**Phonemes related metrics** assess efficiency and clarity of phonetic articulation in speech by measuring the ratio of phonation time to the number of syllables [144] • Phonation to Syllable Ratio (PHONATION_TO_SYLLABLE): This metric, calculated using both Whisper and PocketSphinx technologies, represents the ratio of phonation duration to the number of syllables spoken. It provides insights into speech fluency and the dynamics of language delivery
**Speech Production Dynamics**
**Speech length metrics** quantifies the total duration of speech segments and Total Locution Time (TLT), which measures the total speech time, including pauses between speech segments. These metrics provide insights into the overall length and pacing of speech production [78, 83, 142] • Phonation Time (PHONATION_TIME): The total duration of voiced segments where the speaker is actively producing sound within a speech sample • Total Length of Talk (TLT): The duration of active speech (excluding pauses at the beginning and end of the audio file) relative to the total length of the audio file, indicating the proportion of time spent talking. [78, 83, 142]
**Token count (**COUNT_TOKENS) measures the total number of tokens uttered across all utterances, providing insights into the dynamics of speech production
**Sentence related metrics** analyzes speech production dynamics by measuring the proportion of each utterance’s duration to the total speech duration (Relative Sentence Duration) and the average number of tokens per sentence (Mean Length of Sentence) [78, 83, 142] • Relative Sentence Duration (RELATIVE_SENTENCE_DURATION): Using Whisper X and Whisper for sentence detection, this metric measures the duration of each sentence relative to the total speech duration, highlighting variations in sentence length across a speech sample • Mean Words in Utterance (MEAN_WORDS_IN_UTTERANCE) measures the average number of words found in each utterance, defined as a continuous block of speech bordered by two pause segments. This metric assesses the complexity and structure of spoken communication • Mean Sentence Length (MEAN_LENGTH_SENTENCE) calculates the average number of words per sentence within a speech sample, providing insights into sentence complexity and linguistic fluency

**Table 8:** Details of statistical functions

Statistical functions
**sma** applies a simple moving average (SMA) smoothing filter with window length 3
**de** calculates the delta (first order derivative) of the contour
**maxPos** is the absolute position of the maximum value
**minPos** is the absolute position of the minimum value
**amean** is the arithmetic mean of the contour
**span** is the contour range ‘max–min’
**linregc1** is the slope (m) of a linear approximation of the contour
**linregc2** is the offset (t) of a linear approximation of the contour
**linregerrA** is the linear error computed as the difference of the linear approximation and the actual contour
**linregerrQ** is the quadratic error computed as the difference of the linear approximation and the actual contour
**stddev** is the standard deviation of the values in the contour
**skewness** is the skewness (3rd order moment) of the values in the contour
**kurtosis** is the kurtosis (4th order moment). of the values in the contour
**quartile1** is the first quartile (25% percentile)
**quartile2** is the second quartile (50% percentile)
**quartile3** is the third quartile (75% percentile)
**iqr1-2** calculates the inter-quartile range: quartile2-quartile1
**iqr2-3** calculates the inter-quartile range: quartile3-quartile2
**iqr1-3** calculates the inter-quartile range: quartile3-quartile1
**percentile1.0** is outlier-robust minimum value of the contour, represented by the 1% percentile
**percentile99.0** is the outlier-robust maximum value of the contour, represented by the 99% percentile
**pctlrange0-1** is the outlier robust contour range ‘max–min’ represented by the range of the 1% and the 99% percentile
**upleveltime75** calculates the percentage of time the contour is above (75% * range + min)
**upleveltime90** calculates the percentage of time the contour is above (90% * range + min)

## Appendix E. Feature selection methods

For identifying the most informative features, we employed four different feature selection/extraction strategies: JMIM [91], LASSO [93], LassoNet [92] and PCA [145].

### JMIM

JMIM, a recently introduced feature selection method, is particularly effective for high-dimensional datasets with small sample sizes. It belongs to the family of joint mutual information (JMI)-based methods. In information theory, mutual information (MI) quantifies how much information one random variable (X) provides about another (Y) by measuring the reduction in the entropy of Y when X is known:

$$I\left( {X;Y} \right) = E\left( Y \right) - E\left( {Y|X} \right)$$

where $E\left(Y\right)$ represents the entropy of the random variable $Y$, defined as follows:

$$E\left( Y \right) = - \mathop \sum \limits_{i = 1}^{N} p\left( {y_{i} } \right)\log \left( {p\left( {y_{i} } \right)} \right)$$

$E\left(Y|X\right)$ represents the conditional entropy, which measures the remaining uncertainty in $Y$ after knowing $X$. It is always less than or equal to the entropy of either variable individually. Conditional entropy is defined as follows:

$$E\left( {Y|X} \right) = { } - \mathop \sum \limits_{j = 1}^{M} \mathop \sum \limits_{i = 1}^{N} p\left( {x_{i} ,{ }y_{j} } \right)\log (p(y_{j} |x_{i} ))$$

The information gain method is the simplest MI-based feature selection approach, assuming that features are conditionally independent. To address this limitation, the JMIM method considers potential dependencies within the feature set $F= \left\{{f}_{1}, {f}_{2}, \dots , {f}_{N}\right\}$ by selecting a subset $S= \left\{{s}_{1}, {s}_{2}, \dots , {s}_{K}\right\}$ where $K$ (number of features in $S$) is less than $N$ (number of features in $F$) and $S\subseteq F$. JMIM aims to minimize redundancy among selected features while maximizing the joint mutual information between the subset $S$ and the outcome class label $Y$. Mathematically, this is expressed as:

$$f_{JMIM} = \arg \mathop {\max }\limits_{{f_{i} { } \in { }F - S}} \left( {\mathop {\min }\limits_{{f_{s} { } \in { }S}} \left( {I\left( {f_{i} ,{ }f_{s} ;Y} \right)} \right)} \right)$$

### Lasso

Lasso (Least Absolute Shrinkage and Selection Operator) is a regularization technique commonly employed in machine learning models to mitigate overfitting and enhance generalization by penalizing large model coefficients. A distinctive feature of Lasso is its capability for automatic feature selection. This is achieved by introducing a penalty term into the objective function, which is proportional to the sum of the absolute values of the coefficients. As a result, the penalty encourages some coefficients to shrink to zero, thereby effectively excluding the associated features from the model.

When Lasso regularization is applied to a classification model, such as a Linear Support Vector Machine (SVM), the model’s objective becomes the minimization of a loss function augmented by the L1 regularization term. Specifically, by combining the SVM’s hinge loss with the L1 regularization, the objective function can be represented as:

$$\mathop {\min }\limits_{{\beta_{0} ,\beta }} \left[ {\frac{1}{n}\mathop \sum \limits_{i = 1}^{n} \mathop {\max }\limits_{{/phantom{i}}} (0,1 - y(\beta_{0} - \mathbb{x}_{i}^{T} \beta )) + \lambda \mathop \sum \limits_{j = 1}^{p} \beta_{j} } \right]$$

The second term is the L1 regularization penalty that encourages sparsity by shrinking some coefficients ${\beta}_{j}$​ to zero. The regularization parameter $\lambda$ controls the trade-off between minimizing the hinge loss and enforcing sparsity.

### LassoNet

LassoNet builds on the principle of LASSO, which uses an L1 penalty to promote sparsity, and extends this concept to neural networks. Specifically, LassoNet integrates the L1 penalty within a multilayer perceptron (MLP) framework by introducing a linear skip connection that links the input features directly to the output. The L1 penalty is applied to the weights of this skip connection, encouraging non-essential feature weights to shrink to zero. A feature is permitted to have non-zero weights in any hidden layer only if its corresponding skip-layer connection retains a non-zero value, a condition enforced through a hierarchical soft-thresholding mechanism.

The objective function of LassoNet is defined as:

$$\mathop {{\mathrm{minimize}}}\limits_{{\theta ,{ }W}} L\left( {\theta ,{ }W} \right) + \lambda \left\| {\theta} \right\|_{1}$$

subject to

$$\left\| {W_{j}^{(1)} } \right\|_{\infty } \le M|\theta_{j} |,for\;each\; feature\;j$$

where $L\left(\theta , W\right)$ is the loss function, $\theta$ represents the skip-layer weights, and ${W}_{j}^{(1)}$ are the weights of the first hidden layer associated with feature $j$. The constraint ties the sparsity of the network’s weights to the sparsity of the linear (skip-layer) components, effectively reducing the number of active features while maintaining model performance.

## PCA

Principal Component Analysis (PCA) is a linear dimensionality reduction technique commonly used in machine learning. It transforms a set of potentially correlated features into a new set of uncorrelated variables by projecting the data onto a new coordinate system. In this system, the directions along which the data’s variation is maximized—called principal components—are identified. These components are orthogonal to one another, ensuring that each subsequent component captures a distinct aspect of the variation not explained by previous components. The first principal component captures the most variance.

Given a data matrix $\mathrm{X}$ of size $n\times p$, where $n$ is the number of samples and $p$ is the number of features, the steps for performing PCA are as follows. First, the data is standardized by subtracting the mean of each feature from the dataset, resulting in a centered data matrix ${\mathrm{X}}_{centered}=X- \mu$, where $\mu$ represents the mean of each feature. Next, the covariance matrix $C$ of the centered data is computed using the formula $C= \frac{1}{n-1}{X}_{centered}^{T}{X}_{Centered}$. The principal components are then determined by finding the eigenvalues $\lambda$ and corresponding eigenvectors $v$ of the covariance matrix, which satisfy the equation $Cv=\lambda v$. These eigenvalues are sorted in descending order, and the top $k$ eigenvectors are selected to form a matrix ${V}_{k}$. Finally, the original data is projected onto these top $k$ eigenvectors to obtain the reduced feature set:

$$X_{reduced} = X_{centered} V_{k}$$

**Table 9:** Hyperparameters tuned for machine learning classifiers

ML classifier	Hyperparameters
Random Forest	params = { n_estimators: [10, 100, 500, 1000], max_features: [2, 4, 6, 8], criterion: ["gini", "entropy"], }
Extra Trees	params = { min_samples_split: [2, 5, 15], max_features: [1, 5, 10], n_estimators: [50, 500, 1000, 5000], }
AdaBoost	params = { n_estimators: [500, 1000, 2000, 5000], learning_rate: [0.001, 0.01, 0.1], }
XGBoost	params = { min_child_weight: [1, 5, 10], gamma: [0.5, 1, 1.5, 2, 5], subsample: [1.0, 0.8, 0.6], colsample_bytree: [0.6, 0.8, 1.0], max_depth: [3, 4, 5], }
SVM	params = { kernel: ["poly", "rbf", "linear"], degree: [2, 4, 8], gamma: ["scale", "auto"], }
MLP	params = { number of neurons: [300, 350, 400, 450, 500, 550, 600] learning rate: [0.001, 0.01, 0.1], number of hidden layers: [1, 2, 3] }

## Code availability

The codes used for data analysis is publicly available here: https://github.com/SpeechCARE/SpeechDETECT-Toolkit.
